# High-Density Papertronics via Laser-Written Hydrophilicity on Hydrophobic Parchment Paper

**DOI:** 10.1021/acsami.6c03065

**Published:** 2026-04-16

**Authors:** Zahra Rafiee, Ruohan Zhang, Seokheun Choi

**Affiliations:** † Bioelectronics & Microsystems Laboratory, Department of Electrical & Computer Engineering, State University of New York at Binghamton, Binghamton, New York 13902, United States; ‡ Center for Research in Advanced Sensing Technologies & Environmental Sustainability, State University of New York at Binghamton, Binghamton, New York 13902, United States

**Keywords:** Papertronics, Paper-based electronics, Sustainable electronics, Parchment paper, Laser-induced hydrophilic patterning, Printed analog circuits

## Abstract

High-resolution paper-based electronics are fundamentally limited by uncontrolled ink spreading within porous cellulose networks, which constrains device density and functional integration. Here, we introduce a laser-induced hydrophilic patterning strategy on commercially available hydrophobic parchment paper that fundamentally redefines the resolution, scalability, and design freedom of papertronics. Local laser modification converts selected regions into ink-guiding hydrophilic microchannels, enabling deterministic confinement of functional materials without wax, masks, or high-temperature processing. This strategy supports few-hundred-micrometer-scale patterning, achieving a > 200% reduction in device footprint relative to wax-based approaches and offering a clear route toward further miniaturization via optical refinement. Using this platform, we realize fully printed resistors, low-loss interconnects, interdigitated capacitors, and integrated low- and high-pass RC filters within a single paper layer, exhibiting predictable, tunable electrical behavior consistent with circuit theory. Importantly, the predominantly cellulose-based substrate preserves biodegradability and disposability, while optional elastomeric encapsulation confers environmental robustness without compromising performance. By unifying high-resolution patterning, functional integration, and environmental compatibility, this work establishes laser-patterned parchment paper as a scalable and sustainable electronics platform, bridging the gap between laboratory papertronics and deployable electronic systems.

## Graphical abstract

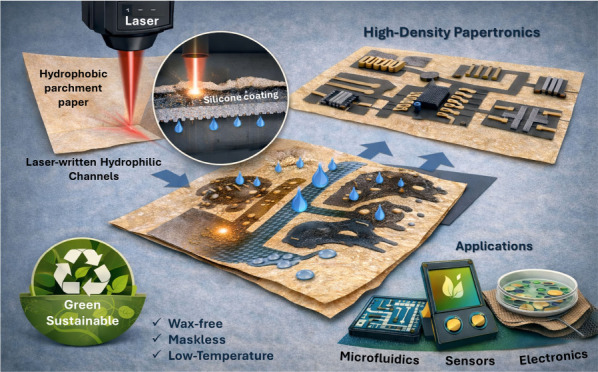

## 1 Introduction

As electronic systems continue to permeate every aspect of modern life, the demand for devices that are lighter, thinner, mechanically compliant, and environmentally responsible has intensified.^1^ Meeting these requirements has driven a paradigm shift in both materials and fabrication strategies. The emergence of two-dimensional materials, including transition-metal dichalcogenides, MXenes, and graphene, has fundamentally altered how electrical, mechanical, and chemical functionalities can be engineered at ultrathin scales.^2^ At the same time, advances in printing-based and additive-manufacturing technologies have enabled electronics to move beyond rigid, wafer-based formats toward flexible, deformable, and large-area architectures.^1,3^ Critically, these approaches support low-temperature, materials-efficient fabrication, offering a viable pathway toward scalable and sustainable electronics without sacrificing performance.^1^

Within this landscape, paper-based materials have emerged as a compelling substrate for next-generation flexible electronics.^4,5^ Beyond their exceptional affordability and global availability, paper offers a unique combination of attributes critical for sustainable and disposable systems: low weight, mechanical flexibility, compatibility with roll-to-roll manufacturing, and facile surface modification through simple printing or postprocessing techniques.^6^ Importantly, the rapidly increasing demand for single-use, transient, and short-lived electronic devices—driven by expanding needs in point-of-care diagnostics, environmental monitoring, food-safety testing, and logistics and shipment tracking—has intensified the search for substrates that are not only inexpensive and scalable but also environmentally benign.^7,8^ In this context, paper’s biodegradability and straightforward postuse handling distinguish it from conventional silicon- and polymer-based platforms, enabling large-scale deployment of electronic devices in applications where long-term durability is unnecessary and material waste must be minimized.

This field has evolved under the banner of paper-based electronics, or papertronics.^9–12^ The term follows the substrate-centric naming convention widely adopted in adjacent communities—analogous to “stretchable electronics,” “e-textiles,” and “printed electronics”—and reflects the fact that paper is not merely a passive carrier but actively participates in device function through its porosity, capillary action, foldability, and biodegradability, imposing a distinct set of fabrication constraints and application opportunities. Papertronics has long been envisioned as a promising pathway toward next-generation green electronics, particularly in response to escalating global concerns over pollution and electronic waste.^9–12^ Despite this vision, the commercial impact of papertronics has remained largely confined to simple, passive, and low-precision components such as radio frequency identification (RFID) and near-field communication (NFC) tags, where packaging and logistics industries prioritize low cost, high throughput, and disposability over functional complexity.^12–15^ The limited scope of adoption reflects a fundamental performance ceiling imposed by conventional paper substrates. The rough, heterogeneous pore networks of cellulose paper undermine patterning fidelity and device uniformity, often necessitating additional coatings, fillers, or calendaring steps to improve smoothness, material adhesion, and functional stability.^6,9,16,17^ However, these compensatory processes frequently involve noneco-friendly materials and energy-intensive fabrication, directly contradicting the sustainability-driven motivations underlying papertronics. As a result, only the simplest device architectures—typically relying on coarse electrode deposition—have achieved practical viability. Compounding these challenges, paper-based devices are intrinsically vulnerable to ambient humidity, mechanical damage, and environmental exposure, which collectively induce performance drift and limit operational stability over time.^18^ While such variability is acceptable for RFID and NFC applications focused on packaging and shipment tracking, it poses a critical barrier to more advanced, tightly specified electronic functions. Although a wide range of electronic components and systems—including printed circuit boards (PCBs),^19,20^ discrete devices,^21,22^ batteries,^23,24^ and touch interfaces^25^ —have been reported on “paper,” many of these demonstrations rely on paper-like polymer substrates, heavily coated or calendered printing papers, or laminated composites.^21,26,27^ In such cases, paper serves merely as a passive mechanical support onto which functional layers are deposited, rather than functioning as the primary, unmodified, cellulose-based platform envisioned in true papertronics. Consequently, these approaches bypass the intrinsic material challenges of real paper, limiting their relevance to genuinely sustainable, low-cost, porous, hydrophilic, and environmentally responsible electronic platforms.

A major breakthrough toward mitigating some of the inherent limitations of papertronics arose from the integration of paper-based microfluidics, or paperfluidics, which transform paper’s naturally porous, capillary-active structure into a functional asset.^12,28^ By leveraging intrinsic wicking behavior, paperfluidic systems enable controlled fluid transport without external pumps or power sources—an elegant strategy that converts paper’s porosity from a liability in electronics into an advantage for analytical devices. Consequently, paperfluidic platforms have become powerful tools for low-cost diagnostic assays,^29^ environmental monitoring,^30^ food-safety testing,^31^ and educational laboratory kits,^32^ owing to their accessibility, portability, and ease of fabrication. The first meaningful convergence of papertronics and paperfluidics was demonstrated by the Whitesides group at Harvard University through their “electrofluidic” components, in which a fluidic channel simultaneously functioned as a conductive electrode.^33^ Building on this concept, our group developed hybrid paperfluidic-electronic techniques that exploit paper’s capillary action to wick functional materials into predefined regions, realizing components such as resistors, capacitors, transistors, and multilayer PCBs supporting amplifier and logic circuits.^34–40^

Despite these advances, the reliance on hydrophilic cellulose paper and wax-based patterning imposes inherent limitations on device density and precision (Figure 1a). Wax printing requires thermal melting that causes uncontrolled lateral and vertical spreading, limiting feature sizes to the millimeter scale and circuit dimensions to tens of centimeters.^34,40^ Paraffin-based wax barriers further introduce residues, nonuniformity, and sustainability concerns.^40^ These constraints underscore a fundamental challenge: true high-resolution papertronics cannot be realized on inherently hydrophilic, highly porous cellulose paper using wax printing or related patterning methods.

**Figure 1:**
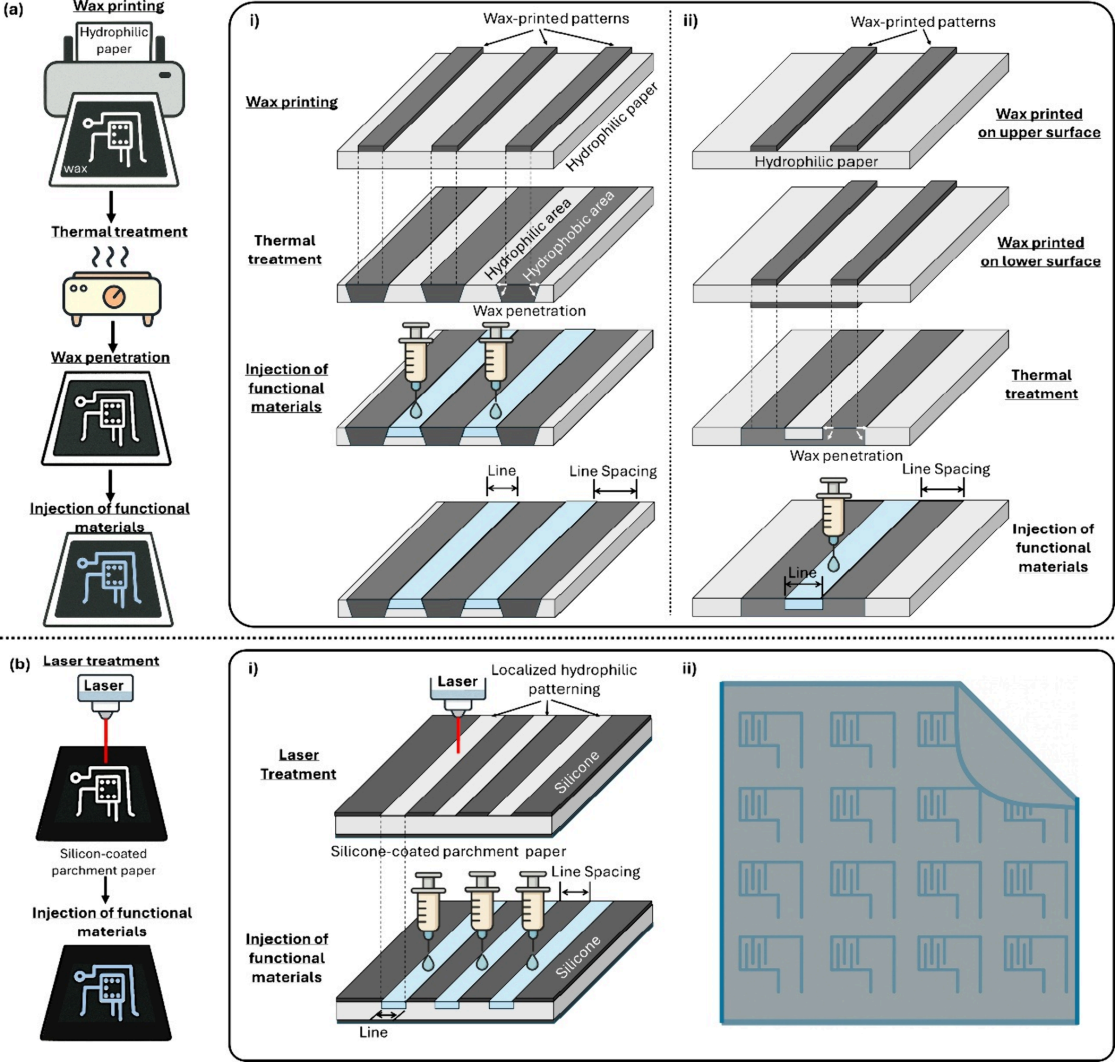
Conceptual illustration of wax-printed versus laser-induced papertronics. (a) Wax-printed papertronics on hydrophilic cellulose paper, highlighting the multiple fabrication steps required for (i) hydrophilic pattern formation via single-sided wax printing and thermal penetration, and (ii) hydrophilic patterning via asymmetric double-sided wax printing and thermal penetration. Molten-wax spreading during thermal penetration inherently blurs feature boundaries and limits the achievable resolution. (b) Laser-induced papertronics on hydrophobic parchment paper, demonstrating a simplified, low-temperature fabrication workflow that enables (i) direct hydrophilic patterning without wax spreading or high-temperature processing, and (ii) the realization of high-density papertronic circuits with narrow hydrophilic channels and tightly spaced insulating regions.

To overcome these limitations, we shift the paradigm by employing hydrophobic parchment paper—a cellulose-based substrate with greatly reduced permeability and minimal spontaneous wicking (Figure 1b). Unlike conventional chromatography paper, parchment maintains structural integrity under low-temperature processing, offers a smoother and more uniform surface, and allows fluid transport to be introduced only when and where it is intentionally patterned. This creates an opportunity for highly controlled and high-fidelity feature definition. Compared with wax-printed papertronics, hydrophobic coating materials such as silicone or plant-derived oils are typically more biocompatible, more sustainable, and more environmentally compatible.^41,42^ Moreover, these coatings form ultrathin layers—typically ∼ 0.5–2 μm thick—on the cellulose substrate. Building upon this substrate advantage, we introduce a laser-written hydrophilicity technique that converts selected regions of hydrophobic parchment into hydrophilic microdomains with high spatial precision. Laser-induced localized heating modifies the surface chemistry and microstructure without requiring additional chemical coatings, fillers, or high-temperature treatments. Critically, these laser-defined hydrophilic features exhibit no lateral spreading, enabling minimum feature sizes and spacings that approach the theoretical resolution limit of the optical system. This strategy enables unprecedented high-density patterning, excellent pattern reproducibility, and device-scale miniaturization—representing a substantial leap beyond what is achievable with wax printing or traditional paperfluidic techniques. Here, we demonstrate fully printed resistors, interconnects, capacitors, and integrated RC filters on laser-treated parchment paper, achieving a > 200% reduction in device footprint relative to wax-based methods. All functional inks employed in this work are water-processable and free of toxic organic solvents or nonbiodegradable metallic components, maintaining material-level sustainability consistent with the environmental objectives of papertronics. Crucially, this improved patterning fidelity enables a materials-programmable design paradigm in which component values—spanning tens of ohms to kilo-ohms for resistors and microfarads to millifarads for capacitors—are tuned entirely through ink formulation without modifying device geometry. PDMS encapsulation further extends device robustness against environmental exposure.

## 2 Results and discussion

### 2.1 Resolution limits: wax-printed versus laser-induced papertronics

In our previous wax-printed papertronics,^34,40^ the geometry of the printed paraffin wax defines the insulating line spacing (*W*_*wax*_), whereas the conductive traces deposited within the residual hydrophilic channels define the functional circuit line width (*W*_*Line*_) (Figure 1a). Because the wax acts as the spacing element rather than the line itself, the effective PCB resolution, dictated jointly by *W*_*wax*_ and *W*_*Line*_, is ultimately constrained by the precision of defining both the hydrophilic channels and the wax barriers. After printing onto hydrophilic cellulose paper, paraffin wax must be melted to form a continuous, leak-free hydrophobic barrier (Figure 1a-i).

During heating, the molten wax wicks through the paper thickness according to the Washburn capillary penetration model, where the penetration distance (*L*) increases with the square root of time (*t)* as

1 $$L=S\sqrt{t}$$

and the spreading coefficient $S=\sqrt{\gamma d/4\eta }$ depends on the surface tension (γ), effective pore diameter (*d*), and viscosity of the molten wax (η).^43^ For typical chromatography papers with a thickness of ∼ 180 μm and a spreading coefficient *S* = 240 μm·min^–1/2^, full through-thickness penetration requires approximately 0.6 min at 120 °C.^43^ However, the molten wax does not move only vertically; it also spreads laterally by approximately 2*L* on each side during this time (Figure 1a-i).^44^ Consequently, even a printed line (*W*_*wax*_) as narrow as ∼ 100 μm inevitably expands to a final width of 400–500 μm once fully melted and penetrated. Simultaneously, *W*_*Line*_ decreases by 2L from its original design, degrading circuit fidelity and functional reliability. This thermally driven transformation imposes a strict physical limit on the minimum line width and spacing achievable with wax-based patterning. To theoretically preserve even a 100 μm conductive line after wax melting, the initial hydrophilic channel must be on the order of ∼ 500 μm, and the insulating wax barrier broadens to a comparable width. This corresponds to a practical PCB resolution of ∼ 0.6 mm (≈100 μm line/≈500 μm line spacing), representing roughly a 3-fold degradation relative to the nominal 100 μm design rule. However, this estimate assumes that sufficient wax volume is available to fully penetrate the paper thickness. In practice, an additional and often overlooked constraint arises from the required wax mass:^45^ narrow printed wax lines simply do not contain enough material to saturate the entire thickness, resulting in incomplete barriers, midlayer pinholes, and leakage pathways (Figure 1a-i). To compensate, wax lines must be intentionally printed thicker and wider, and in many cases, asymmetric double-sided wax printing becomes necessary to deliver adequate wax volume (Figure 1a-ii). These adjustments further increase the effective spacing width and introduce added uncertainty in the final patterned dimensions. Thus, the resolution limit is determined not by the nominal printer resolution but by the coupled requirements of sufficient vertical wax penetration to form reliable barriers, unavoidable lateral spreading during melting, and the need for adequate wax volume. For these reasons, our previous papertronic platform conservatively set the line spacing to 0.5–1.0 mm—even though the printer itself could pattern much narrower features.^34,40^ Ultimately, the experimentally achievable PCB resolution for wax-based papertronics was approximately 0.9–1.5 mm pitch, defined by ∼ 0.4–0.5 mm wax line width (*W*_*Line*_) and ∼ 0.5–1.0 mm spacing (*W*_*wax*_), substantially above the theoretical minimum.^40^ Asymmetric wax printing reduces the required thermal treatment time and the amount of molten wax, allowing the initial printed geometry to be better preserved (Figure 1a-ii).^46^ However, even this localized patterning sacrifices resolution and introduces additional complexity: asymmetric wax deposition demands precise front-to-back alignment, carefully controlled heating conditions, and strict process steps to ensure consistent partial penetration. These requirements limit its practicality and still prevent the formation of high-resolution features.

The laser-induced approach inverts the conventional paradigm (Figure 1b): rather than patterning hydrophobic barriers on hydrophilic paper, we selectively expose hydrophilic domains within uniformly hydrophobic parchment. The CO_2_ laser selectively decomposes the sub-2-μm silicone layer,^47^ converting irradiated regions into hydrophilic microdomains without any molten phase or capillary-driven spreading. While seminal, that work was confined to fluid manipulation. Here, we build upon—and fundamentally extend—this principle by repurposing laser-induced hydrophilicity as a high-resolution patterning tool for electronic architectures. In contrast to wax-based methods, this approach involves no molten phase, no vertical material transport, and no reliance on capillary spreading to define feature boundaries. Instead, pattern resolution is governed by the optical spot size and local thermal diffusion length, decoupling feature definition from fluid dynamics. As a result, hydrophilic patterns can be generated with micrometer-scale precision using a streamlined two-step workflow: (i) laser writing of hydrophilic domains, followed by (ii) targeted injection of functional materials (Figure 1b-i). This paradigm enables deterministic, high-density papertronics that were previously inaccessible using conventional paper-based fabrication strategies.

The patterning capability is precisely regulated by tuning the laser power and scan speed. For the VLS 3.50 laser system used in this work, equipped with a 50 W CO_2_ tube, the laser power and scan speed were systematically tuned to optimize local heating and patterning of silicone-coated parchment paper (Figure S1a). At high power and low scan speed, the laser overheated the substrate, burning through the hydrophilic cellulose layer and cutting the paper. At low power and high speed, the silicone coating was only partially removed—or not removed at all—preventing proper penetration of the functional materials. The optimized operating condition for strait-line patterning was identified at laser power levels below 12% (corresponding to a delivered power of 6 W) and 100% scan speed (1.27 m/s). Using the standard 2″ focusing lens, the laser beam is theoretically focused to a ∼ 127 μm diameter, yielding a dwell time of ∼ 100 μs at the optimized scan speed. Because organic materials typically absorb roughly 50–100% of CO_2_ laser radiation,^48,49^ we conservatively assume an absorptance of 50%. The absorbed energy during one dwell event is therefore *E*_abs_ = 0.5*P t* = 0.3 mJ. If we approximate the laser-affected volume as a cylinder with cross-sectional area *A* = π­(*d*/2)^2^ ≈ 1.27 × 10^–8^m^2^ and thickness *h* ≈ 75 μm (the parchment thickness), the heated volume is *V* ≈ 9.5 × 10^–13^m^3^. Taking the effective density and specific heat of the parchment to be ρ ≈ 800 kg·m^3^ and *c* ≈ 1400J·kg^–1^·K^–1^, the mass of the heated voxel is *m* = *ρV* ≈ 7.6× 10^–10^kg, and the estimated temperature rise is Δ*T* ≈ *E*_bs_ /(*mc*)≈ 280 K. This places the local peak temperature in the range of 300 °C,^50^ which is sufficient to decompose the silicone coating and modify the top cellulose surface, yet below the catastrophic ablation threshold.^51^ This quantitative analysis confirms the physical validity of our experimentally optimized laser parameters. The lateral extent of the modified region is dictated by the laser spot size and thermal diffusion in the paper. The thermal diffusion length (*L*_*th*_) during the dwell time (*t*) is $L_{\mathrm{th}}\approx 2\sqrt{\alpha t}$ , with cellulose diffusivity α ≈ 1 × 10^–7^m^2^·s^–1^,^52^ giving *L*_th_ ≈ 6 μm, far smaller than ∼ 127 μm beam diameter. Thus, lateral heat spread is negligible, and the hydrophilic modification remains confined essentially within the optical footprint of the beam. With the Gaussian intensity profile of the beam—of which approximately 50–70% of the radius contributes effectively to silicone removal—we can theoretically generate hydrophilic channels ∼ 60–80 μm wide while preserving hydrophobic spacing of ∼ 80–120 μm without unintended bridging. This corresponds to a PCB pitch of ∼ 140–200 μm, representing an approximately 3-fold improvement over the theoretical (∼600 μm) resolution limit of wax-printed papertronics, and enabling substantially higher-density electronic architectures (Figure 1b-ii). This enhanced patterning resolution is the critical enabler for the functional demonstrations that follow: the precise ink confinement afforded by laser-defined channels underpins the broad electrical tunability of individual components (Section 2.3) and the compact integration of multicomponent analog circuits (Section 2.4) that would not be realizable at the resolution limits of wax-based methods.

### 2.2 Laser-Driven Surface Transformation and Guided Functional Material Deposition

To examine whether this theoretical resolution could be realized in practice, laser patterning was evaluated across a range of line widths and spacings (Figure S1b and S1c). While laser patterning alone was capable of achieving pitches approaching ∼ 200 μm, the ultimate resolution was determined after functional ink deposition (Figures 2a and 2b). Functional inks used to define conductive traces and electronic components, such as poly­(3,4-ethylenedioxythiophene):poly­(styrenesulfonate) (PEDOT:PSS), possess controllable finite viscosity and density, which result in limited lateral spreading within the three-dimensional cellulose fiber network, typically on the order of micrometers.^33^ Notably, because silicone-coated parchment paper features a denser fiber structure and smaller intrinsic pore size than conventional filter paper (Figure 2c), the laser-activated hydrophilic regions are expected to exhibit locally compacted surfaces with reduced effective pore sizes, further constraining ink propagation and contributing to the observed enhancement in pattern fidelity. Although laser patterning can define minimal pitches, adjacent conductive lines must remain electrically isolated during conductivity testing, confirming that ink spreading does not extend beyond the laser-defined hydrophilic channels (Figure S2). Under these practical constraints, we achieved conductive lines with a width of ∼ 250 μm and a line spacing of ∼ 300 μm (Figure 2b), corresponding to a PCB pitch of ∼ 550 μm. This resolution compares favorably not only with wax-based papertronics (0.9–1.5 mm pitch)^40^ but also with inkjet-printed and screen-printed paper electronics, which typically achieve feature sizes of 0.5–2 mm on unmodified cellulose substrates. While lithographic or laser-induced graphite (LIG) approaches can yield finer features,^49^ they require either cleanroom processing or produce carbonized traces with limited material versatility, respectively. The present approach uniquely combines submillimeter resolution with solution-processable, biodegradable functional inks in a single-step, mask-free workflow. Scanning electron microscopy (SEM) and energy-dispersive X-ray spectroscopy (EDS) further confirmed successful ink deposition and the presence of sulfur (S)—a characteristic elemental signature of PEDOT:PSS—within the patterned line regions (Figures 2c and 2d). The reproducibility of the laser-patterning process is further evidenced by the minimal device-to-device variation observed across all component types, including resistors, capacitors, and RC filter cutoff frequencies, as discussed in the following sections. Such consistency underscores the deterministic nature of laser-optical patterning, which eliminates the stochastic spreading associated with wax-based methods.

**Figure 2:**
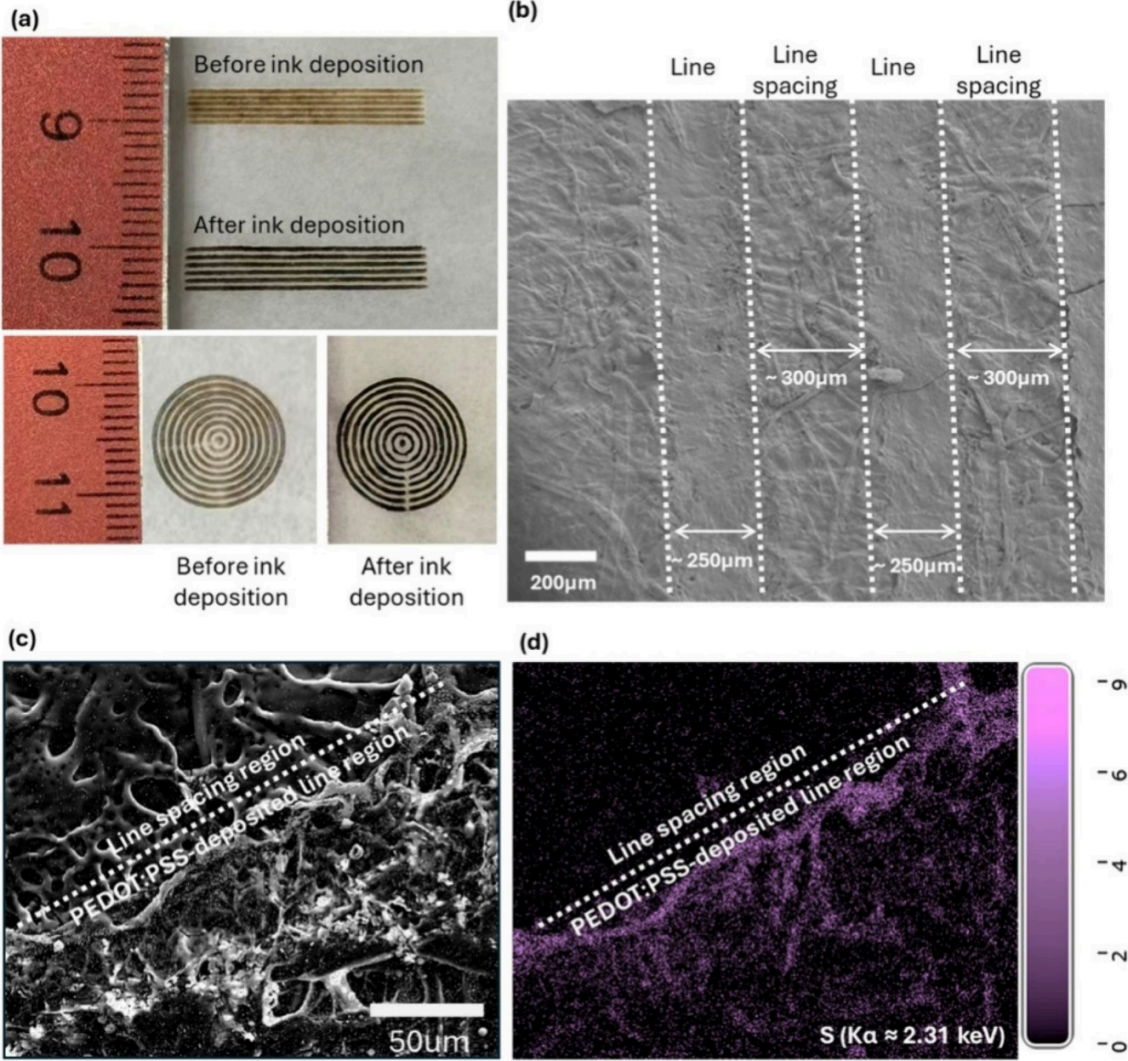
Laser patterning on parchment paper. (a) Laser patterning performed both before and after conductive ink deposition to define and refine conductive features; a centimeter-scale ruler is included to verify feature dimensions. (b) Scanning electron microscopy (SEM) image of a patterned paper-based PCB with straight-line features. Conductive lines were defined by laser treatment followed by PEDOT:PSS ink deposition, while the line spacing corresponds to nonlaser-treated hydrophobic regions. (c) High-resolution SEM image and (d) corresponding energy-dispersive X-ray spectroscopy (EDS) analysis of the conductive line, confirmed by the presence of sulfur (S), and the adjacent line-spacing regions.

Fourier-transform infrared (FTIR) spectroscopy was employed to elucidate the chemical composition of the pristine parchment paper, the surface modifications induced by laser treatment, and the subsequent physicochemical evolution following PEDOT:PSS deposition (Figure S3). The FTIR spectrum of pristine parchment paper reflects its composite, multilayer structure consisting of a cellulose-based fibrous substrate and a surface-applied antistick coating (Figure S3a). A broad absorption band centered at approximately 3350 cm^–1^ is attributed to O–H stretching vibrations, arising predominantly from the extensive hydrogen-bonded hydroxyl groups in cellulose and hemicellulose, with additional contributions from physically adsorbed water. Weak absorption bands near 2900 cm^–1^ correspond to aliphatic C–H stretching vibrations of CH_2_ and CH_3_ groups associated with the cellulose backbone and organic additives. A moderate band at approximately 1650 cm^–1^ is assigned to the H–O–H bending mode of water retained within the porous cellulose network. Importantly, a distinct and sharp absorption peak near 1260 cm^–1^, corresponding to Si–CH_3_ symmetric bending vibrations, serves as a definitive spectral marker for the presence of a PDMS-based silicone coating, which is widely used in commercial parchment paper as a nonstick, hydrophobic surface layer. Additional confirmation of the silicone coating is provided by the strong absorption in the 1000–1100 cm^–1^ region, where Si–O–Si stretching vibrations overlap with the C–O–C and C–O stretching modes of cellulose, and by a peak near 798 cm^–1^, attributed to Si–C stretching and Si–CH_3_ rocking vibrations. A band observed near 1430 cm^–1^ indicates the presence of carbonate (CO_3_^2–^) groups, consistent with the incorporation of calcium carbonate (CaCO_3_) as a filler to enhance opacity, brightness, or thermal stability. Collectively, these features confirm that the pristine parchment paper consists of a hydrophilic cellulose bulk substrate functionalized with a thin, surface-localized silicone coating.

Laser irradiation induces pronounced photothermal and photochemical modifications to the parchment paper surface (Figure S3b). Most notably, the Si–CH_3_ (∼1260 cm^–1^) and Si–O–Si (∼1060 cm^–1^) features associated with the silicone coating are significantly diminished or absent, indicating effective removal or decomposition of the silicone layer. This confirms that the laser treatment selectively disrupts the hydrophobic surface coating rather than penetrating through the full paper thickness. Concurrently, substantial changes are observed in the cellulose-related bands. The O–H stretching envelope (3000–3700 cm^–1^) decreases in intensity and broadens, consistent with dehydration and partial degradation of cellulose hydroxyl groups under localized laser heating. New absorption bands emerge in the 1700–1750 cm^–1^ range, which are attributed to C = O stretching vibrations, indicating the formation of carbonyl-containing species such as aldehydes, ketones, or carboxylic acids as a result of thermal–oxidative degradation of cellulose. Additionally, the appearance of a broad feature in the 1580–1600 cm^–1^ region is characteristic of aromatic C = C stretching vibrations, signifying the formation of carbonized, sp^2^-rich structures. This partial carbonization is consistent with intense but spatially confined photothermal heating and confirms that laser treatment converts the surface from an organic polymer composite into an oxygen-functionalized carbonaceous layer. Importantly, this transformation is limited to the near-surface region, preserving the mechanical integrity of the underlying cellulose matrix.

Following deposition of PEDOT:PSS onto the laser-treated surface, the FTIR spectrum undergoes a pronounced evolution that confirms successful conductive polymer integration (Figure S3c). The overall spectral baseline and signal intensity recover toward those observed in the pristine state, indicating that the PEDOT:PSS film uniformly coats and planarizes the laser-modified surface, thereby reducing scattering effects associated with surface roughness and carbonization. The 3000–3700 cm^–1^ region exhibits a renewed increase in intensity, now dominated by O–H stretching vibrations of sulfonic acid (−SO_3_H) groups in the PSS component and strongly bound water molecules, reflecting the highly hydrophilic nature of the PEDOT:PSS layer. The band near 1650 cm^–1^ becomes more pronounced, primarily due to H–O–H bending vibrations of water retained within the polymer film. Most definitively, the fingerprint region (600–1000 cm^–1^) displays increased complexity and intensity, consistent with PEDOT:PSS deposition. Characteristic features include aromatic C–H out-of-plane bending modes associated with the PSS backbone and contributions from sulfonate S = O stretching vibrations, typically appearing as strong features near 1125 and 1035 cm^–1^. These sulfonate-related bands provide unequivocal evidence of PSS incorporation and confirm the presence of the PEDOT:PSS conductive network on the paper substrate.

### 2.3 Paper-Based Electronic Components

Building on laser-induced hydrophilic patterning, the controlled selection of functional inks and device architectures enables the deterministic formation of electronic components directly on hydrophobic parchment paper. Functional materials are confined exclusively within laser-defined hydrophilic regions, allowing resistive, conductive, and capacitive elements to be precisely defined without lateral spreading or electrical interference. This strategy enables the integration of paper-based resistors, interconnects, and charge-storage components while preserving the hydrophobic insulation of the surrounding substrate. Together, surface-selective laser modification and ink-guided self-alignment establish a robust platform for high-resolution, multifunctional papertronic circuits with broad material compatibility.

#### 2.3.1 Resistors

Paper-based resistors were fabricated by depositing PEDOT:PSS into laser-defined hydrophilic channels on parchment paper. Figure 3 presents the overall design strategy, morphology, and electrical tunability of the laser-defined paper resistors. As schematically illustrated in Figure 3a, resistor values can be independently controlled through three parameters: ink concentration, dimethyl sulfoxide (DMSO) content, and resistive line length. The laser-defined hydrophilic channels act as deterministic templates that confine conductive inks, while the surrounding silicone-coated parchment remains hydrophobic and electrically insulating. This spatial confinement decouples electrical tuning from uncontrolled ink spreading and penetration, enabling predictable and reproducible resistor geometries.

**Figure 3:**
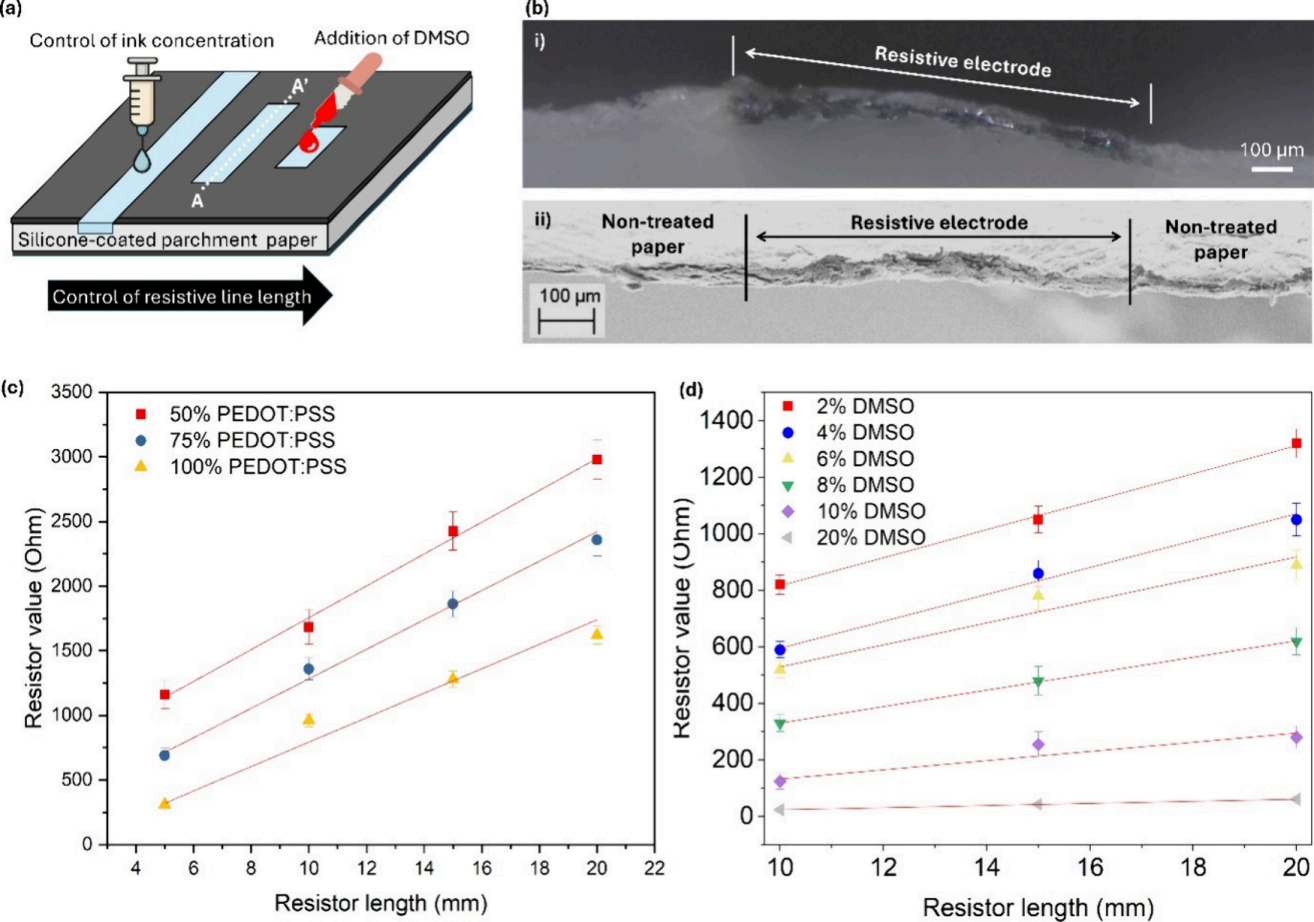
Paper-based Resistors. (a) Schematic illustration of laser-defined paper resistors tuned by three independent parameters: ink concentration, DMSO addition, and resistor geometry. (b) (i) Optical microscopy image and (ii) SEM image of the A–A′ cross-section of a representative resistor, showing that PEDOT:PSS is precisely confined within the laser-treated hydrophilic region and surrounded by untreated hydrophobic parchment paper. (c) Measured resistance of resistors fabricated with varying ink volumes, where PEDOT:PSS was systematically diluted with deionized water to concentrations of 100%, 75%, and 50%, plotted as a function of resistor length. (d) Resistance as a function of DMSO concentration in the PEDOT:PSS matrix, with weight percentages ranging from 2% to 20%, plotted as a function of resistor length. Data in (c) and (d) represent mean ± standard error from at least 10 independent measurements per data point. Solid lines are least-squares linear fits.

Optical microscopy and SEM cross-sectional images acquired along the A–A′ plane (Figure 3b-i and (ii) reveal that PEDOT:PSS is confined exclusively to the laser-activated hydrophilic channel, with the adjacent parchment regions retaining their hydrophobic and electrically insulating character. The resulting architecture forms a well-defined resistive electrode bounded by sharp hydrophobic interfaces, demonstrating that laser activation effectively replaces wax barriers as the insulating element. Electrical characterization reveals that the resistance of the laser-defined PEDOT:PSS resistors is highly tunable through combined control of ink composition and geometry. Figure 3c shows the measured resistance as a function of resistor length for three PEDOT:PSS concentrations (100%, 75%, and 50% diluted with deionized water). For 800 μm-wide conductive lines, resistance increases monotonically as the line length is extended from 4 to 22 mm for all ink concentrations, consistent with classical resistive scaling. At a fixed length, higher PEDOT:PSS concentration yields lower resistance, reflecting an increased density of conductive pathways within the cellulose fiber network. Notably, the resistance–length relationship remains linear across all ink concentrations, indicating uniform ink confinement and a consistent effective cross-section along the resistive channel—behavior that is difficult to achieve in wax-based systems, where molten wax penetration and ink spreading distort the effective geometry. The linear fits exhibit positive y-intercepts, which are attributed to the combined contributions of contact resistance at the measurement interface and localized ink accumulation at the channel end points. These length-independent resistance components become proportionally more significant for shorter resistors, producing a slight upward deviation from ideal proportional scaling at the shortest measured lengths. For resistors below ∼ 10 mm, a two-parameter model (R = R_0_ + ρ′L, where R_0_ captures the contact and edge-effect contribution) provides a more accurate description than simple proportional scaling. This consideration should be accounted for in device geometry design when targeting specific resistance values at short channel lengths.

Beyond ink concentration and geometry, the incorporation of DMSO as a secondary dopant provides an especially powerful means of fine-tuning resistive behavior. Although DMSO evaporates during drying and is absent from the final device, its presence during film formation induces conformational rearrangement of PEDOT chains and partial removal of excess insulating PSS, yielding persistent improvements in interchain connectivity and charge carrier mobility.^33,36,40^ As shown in Figure 3d, increasing the DMSO content from 2 to 20 wt % produces a pronounced reduction in resistance across all resistor lengths. At low DMSO concentrations (2–4 wt %), resistance increases steeply with length, whereas higher DMSO contents significantly reduce the overall resistance and flatten the resistance–length slope. This behavior is consistent with the secondary doping mechanism described above, in which DMSO-induced morphological changes persist in the dried film and enhance conductivity.^33,36,40^ At high DMSO content, resistance values as low as tens of ohms are achieved even for the longest resistors, demonstrating fine-scale electrical tunability over more than an order of magnitude. Importantly, the resistance remains strongly length-dependent even at high DMSO concentrations, confirming that geometric control is preserved and that electrical tuning does not arise from uncontrolled ink delocalization.

Overall, the resistance of the paper-based resistors can be continuously tuned over a broad range—from approximately tens of ohms to several kilo-ohms—through independent control of ink concentration, DMSO content, and resistive line length. Consistent with the resolution analysis in Section 2.1, the laser-defined resistors achieve a > 200% footprint reduction relative to wax-patterned counterparts.^34,40^

#### 2.3.2 Interconnects

The fabrication of interconnects follows the same laser-defined patterning strategy, but prioritizes minimal sheet resistance and environmental robustness over broad lengths (Figure 4a). To evaluate performance under demanding conditions, long conductive paths with lengths up to a few cm and widths of 800 μm were designed and fabricated as a representative extreme case (Figure 4b). PEDOT:PSS was selected as the base conductive material for interconnects due to its excellent aqueous processability, tunable electrical properties, and strong compatibility with cellulose fiber networks. To further enhance conductivity and mechanical robustness, 5 wt % graphene was incorporated into the PEDOT:PSS matrix. Graphene’s large surface-to-volume ratio and high intrinsic conductivity promote efficient charge transport and reinforce the polymer network, while also improving mechanical and thermal stability.^53^

**Figure 4:**
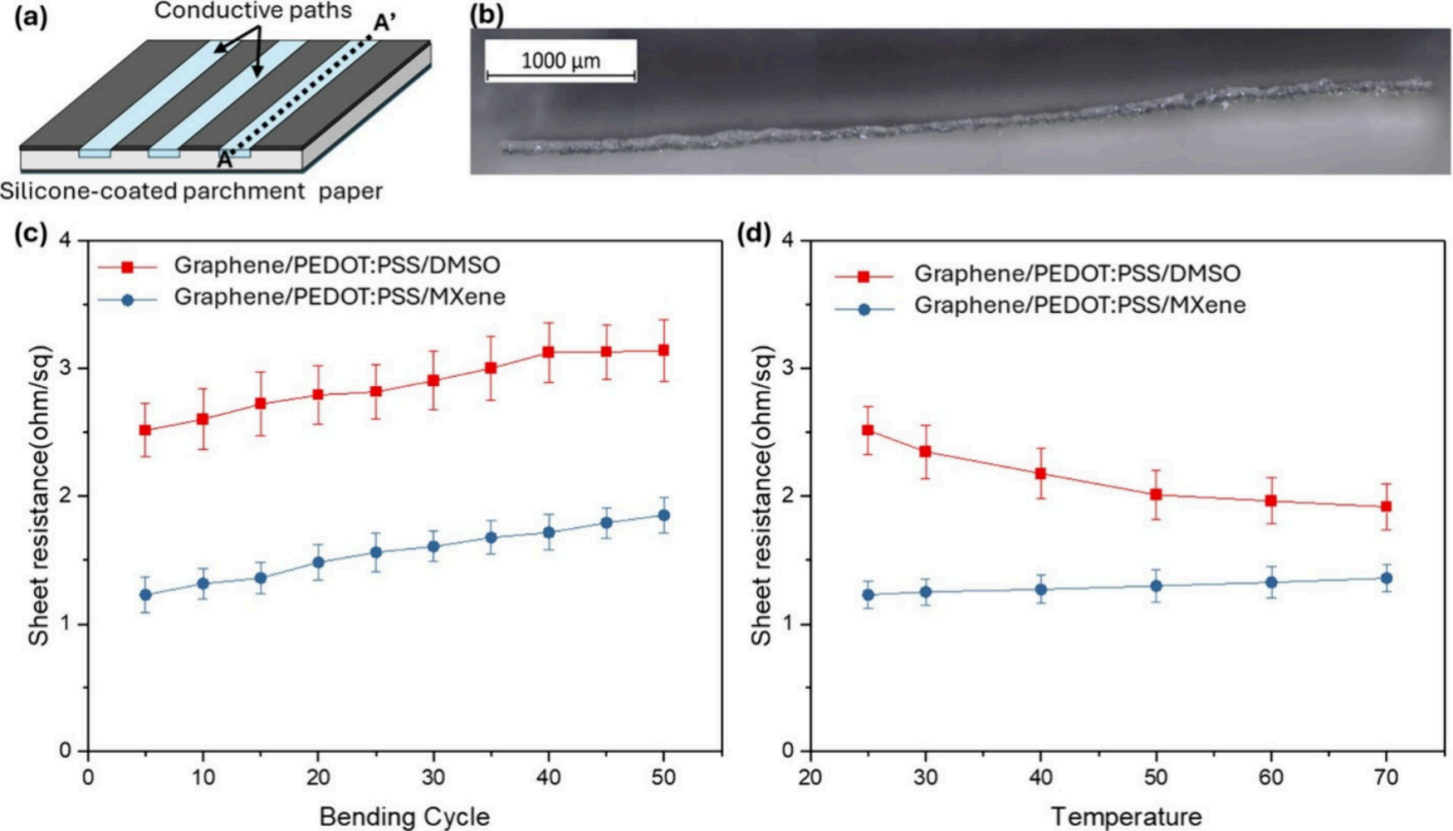
Paper-based Interconnects. (a) Schematic illustration of laser-defined paper interconnects. (b) Optical microscopy image of the A–A′ cross-section of a representative interconnect, showing that the conductive ink is precisely confined within the laser-treated hydrophilic region of the parchment paper. (c) Sheet resistance of interconnects measured over repeated bending cycles, comparing two ink formulations: graphene/PEDOT:PSS/DMSO and graphene/PEDOT:PSS/MXene. The graphene/PEDOT:PSS/MXene ink exhibits superior mechanical endurance, with minimal resistance increase under cyclic bending. (d) Sheet resistance of interconnects measured under elevated temperatures (25–70 °C), demonstrating the enhanced thermal stability of the graphene/PEDOT:PSS/MXene ink, which maintains lower overall sheet resistance across the tested temperature range.

To optimize interconnect performance, two additional formulations were investigated: the incorporation of 20 wt % DMSO or 20 wt % Ti_3_C_2_T_*x*_ MXene. DMSO serves as a secondary dopant that enhances PEDOT:PSS conductivity through the morphological reorganization mechanism described in Section 2.3.1. In contrast, MXene serves as a highly conductive two-dimensional additive that forms percolative pathways within the polymer matrix,^2^ while also improving mechanical endurance and thermal stability due to its metallic conductivity and strong interfacial interactions with PEDOT:PSS and graphene. Figures 4c and 4d compares the sheet resistance stability of paper-based interconnects fabricated using two ink formulations—graphene/PEDOT:PSS/DMSO and graphene/PEDOT:PSS/MXene—under cyclic bending and elevated temperature. Despite the absence of DMSO in the MXene formulation, the MXene-containing interconnects exhibit consistently lower sheet resistance and improved stability, indicating that MXene provides a dominant benefit to charge transport and network robustness in the paper-supported composite. Under repeated bending (Figure 4c), graphene/PEDOT:PSS/DMSO shows a clear and monotonic resistance increase, rising from approximately 2.5 Ω/sq to ∼ 3.1 Ω/sq over 50 cycles (≈25% increase). This gradual drift suggests progressive disruption of conductive pathways during deformation, likely due to strain-induced microcracks, partial debonding from cellulose fibers, and loss of percolation continuity within the polymer/graphene network. In contrast, the graphene/PEDOT:PSS/MXene interconnects maintain substantially lower sheet resistance across all bending cycles, increasing only modestly from roughly 1.2 Ω/sq to ∼ 1.8 Ω/sq. Although the absolute increase is visible, the curve remains well below the DMSO-containing formulation throughout the test, demonstrating that MXene effectively stabilizes electrical conduction under mechanical strain. This behavior is consistent with MXene flakes acting as mechanically robust, two-dimensional conductive bridges that maintain percolation even when the polymer matrix undergoes microstructural deformation.^54^ In addition, MXene’s large aspect ratio and strong interfacial interactions with PEDOT:PSS and graphene can suppress crack propagation and preserve conductive connectivity across fiber junctions during bending.^55^ Further investigation is required to quantitatively elucidate the underlying interfacial bonding mechanisms, strain distribution, and percolation dynamics responsible for this enhanced mechanical resilience. Temperature-dependent measurements further distinguish the two formulations (Figure 4d). For graphene/PEDOT:PSS/DMSO, sheet resistance decreases from about 2.5 Ω/sq at 25 °C to ∼ 1.9 Ω/sq at 70 °C, indicating a relatively strong temperature dependence. This trend is typical of conductive polymer systems, where thermal activation and polymer relaxation can modify charge transport pathways; however, the larger slope implies greater sensitivity of electrical performance to operating temperature. In comparison, graphene/PEDOT:PSS/MXene shows a much smaller temperature dependence, remaining near ∼ 1.2–1.35 Ω/sq across 25–70 °C. The weak slope indicates improved thermal robustness and more stable transport pathways. This can be attributed to MXene’s metallic-like conductivity and its ability to form persistent percolation networks that are less affected by polymer chain mobility or temperature-induced morphological variations.^56^

The exceptionally low sheet resistance values achieved in this work represent a significant advancement over previously reported paper-based conductive materials. In many printed PEDOT:PSS systems without specialized patterning, sheet resistances are often in the tens to hundreds of ohms per square; for example, multilayer PEDOT:PSS films on paper or flexible substrates have been reported with sheet resistances as low as ∼ 29 Ω/sq after solvent or surfactant enhancement, but more commonly fall well above 100 Ω/sq.^57^ Commercial aqueous PEDOT:PSS dispersions also typically exhibit sheet resistances in the 100–1000 Ω/sq range on flexible substrates without additional engineering. While recent studies have explored enhanced papertronics devices incorporating PEDOT:PSS for sensors and energy harvesters, these efforts generally do not achieve sub-10 Ω/sq sheet resistance due to limitations in ink confinement, percolation network formation, and substrate effects.^57^ In contrast, the laser-defined conductive networks developed here, incorporating advanced additive formulations, exhibit sheet resistances on the order of ∼ 1 Ω sq^–1^, representing a one- to two-orders-of-magnitude reduction compared with typical PEDOT:PSS patterns on paper and approaching conductivity levels more commonly associated with treated films in rigid electronic systems.

#### 2.3.3 Capacitors

Achieving wide, controllable capacitance on paper has been hindered by substrate heterogeneity and the complexity of wax-based or vertical device architectures.^40^ In this work, these limitations are overcome by implementing a laser-defined interdigitated capacitor architecture on hydrophobic parchment paper. As illustrated in Figure 5a, conductive PEDOT:PSS electrodes are precisely patterned into interdigitated fingers (each figure: 10 mm in length and 0.8 mm in width) using laser-induced hydrophilic channels, while the surrounding silicone-coated parchment remains hydrophobic and electrically insulating (Figure S4). This planar configuration eliminates the need for through-thickness electrolyte infiltration or asymmetric wax penetration, thereby simplifying fabrication and improving device reproducibility. Following electrode patterning, a poly­(vinyl alcohol) (PVA)-based gel electrolyte was introduced via a laser-fabricated paper stencil, enabling uniform electrolyte deposition across the interdigitated electrode array. Optical microscopy and SEM cross-sectional images taken along the A–A′ plane (Figure 5b-i and (ii) confirm that the PEDOT:PSS electrodes are precisely confined within the laser-activated regions and that the PVA gel electrolyte conformally covers the entire interdigitated structure. This intimate electrode–electrolyte contact is critical for achieving stable and predictable capacitive behavior.

**Figure 5:**
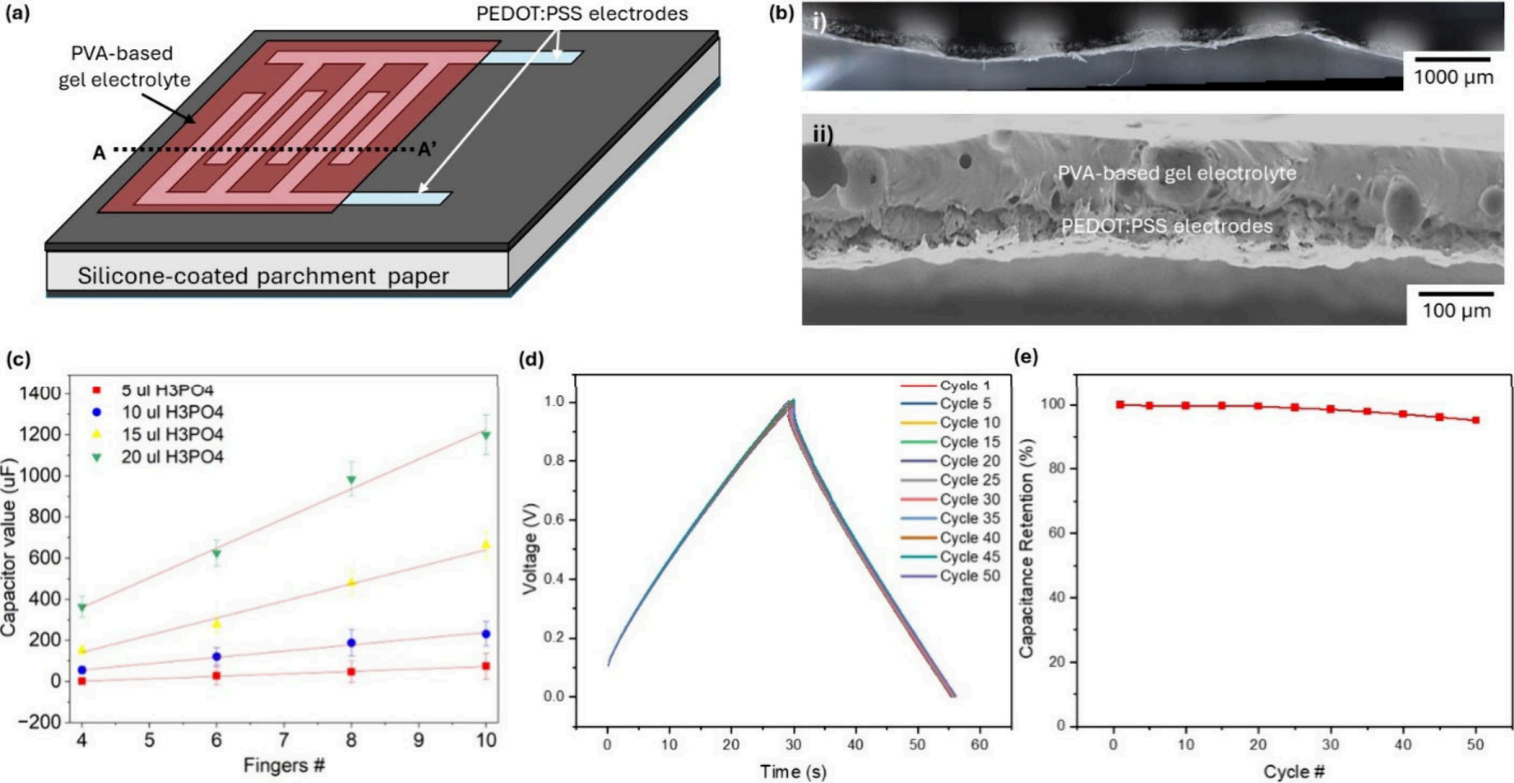
Paper-based Capacitors. (a) Schematic illustration of a laser-defined interdigitated capacitor fabricated on hydrophobic parchment paper. (b) (i) Optical microscopy image and (ii) SEM image of the A–A′ cross-section of a representative capacitor, showing that conductive PEDOT:PSS ink is precisely confined within the laser-treated hydrophilic regions to form interdigitated electrode fingers, while the surrounding parchment paper remains hydrophobic and electrically insulating. A poly­(vinyl alcohol) (PVA)-based gel electrolyte uniformly covers the interdigitated electrodes. (c) Measured capacitance of capacitors fabricated with varying H_3_PO_4_ concentrations in the PVA gel electrolyte, plotted as a function of electrode finger number. Solid lines in (c) are least-squares linear fits. (d) Galvanostatic charge–discharge (GCD) curves measured at a constant current of 2 μA after different numbers of charge–discharge cycles. (e) Capacitance retention as a function of cycling number, demonstrating the cycling stability of the paper-based capacitor.

Figure 5c presents the measured capacitance as a function of electrode finger number for devices fabricated with increasing amounts of H_3_PO_4_ incorporated into the PVA gel electrolyte (5, 10, 15, and 20 μL). For all electrolyte compositions, capacitance increases monotonically with the number of interdigitated fingers, reflecting the enlarged effective electrode surface area and reduced ionic diffusion distance inherent to the interdigitated geometry. More importantly, increasing the H_3_PO_4_ content within the PVA matrix produces a substantial enhancement in capacitance across all device geometries. This behavior arises from the increased ionic concentration and improved proton conductivity of the gel electrolyte,^40^ which facilitates more efficient electric double-layer formation at the PEDOT:PSS/electrolyte interface. At higher acid loadings, the enhanced ionic mobility and reduced internal resistance enable greater charge accumulation, resulting in capacitance values spanning from the low microfarad regime to over 1.2 mF. The ability to modulate capacitance over nearly 3 orders of magnitude using only electrolyte composition and electrode geometry underscores the exceptional tunability of this laser-patterned capacitor platform. The linear fits exhibit positive y-intercepts, which are attributed to baseline double-layer capacitance at the electrode contact pads and interconnect regions, fringing field effects at the terminal fingers, and electrolyte distribution effects at low finger counts. These contributions are independent of finger number and become proportionally less significant as the number of interdigitated fingers increases. The approximately linear scaling observed in the 6–10 finger regime guided the selection of 6-finger geometries for the RC filter circuits in Section 2.4, providing a favorable balance between compact footprint, predictable capacitance, and reliable tunability through electrolyte composition alone.

The electrochemical behavior of the paper-based capacitors was further evaluated using galvanostatic charge–discharge (GCD) measurements. Figure 5d shows representative GCD profiles recorded at a constant current of 2 μA over a voltage window of 0–1 V for a 10-finger device incorporating a PVA gel electrolyte with 20 μL of H_3_PO_4_. The charge–discharge curves exhibit a predominantly linear and symmetric triangular shape, characteristic of capacitive energy storage. A small degree of asymmetry between the charging and discharging branches suggests a combined contribution from electric double-layer capacitance and pseudocapacitive processes associated with the PEDOT:PSS electrodes. Importantly, the minimal voltage drop (IR drop) observed at the onset of discharge indicates a low internal resistance, reflecting efficient ion transport within the gel electrolyte and strong electrode–electrolyte coupling. The capacitance was calculated using

2 $$C=\left(I_{dis}\times \Delta t\right)/\left(\Delta V-\Delta V_{IR}\right)$$

where *I*_*dis*_ is the discharge current, *Δt* is the discharge time, *ΔV* is the total voltage change, and *ΔV*_*IR*_ is the instantaneous IR drop.^58,59^ The calculated capacitance for the first cycle (∼1.2 mF) agrees closely with values obtained via direct multimeter measurements, validating the electrochemical characterization. To assess cycling durability, the GCD process was repeated for 50 consecutive cycles. As shown in Figure 5e, the capacitor retains more than 95% of its initial capacitance after 50 cycles, indicating excellent cycling stability and minimal degradation of the electrode–electrolyte interface.

### 2.4 Paper-based Integrated Circuits

To demonstrate circuit-level integration, first-order low-pass and high-pass RC filters were fabricated on parchment paper (Figure 6). All hydrophilic regions were defined in a single laser step, and components were sequentially deposited using paper stencils. We first implemented a first-order low-pass RC filter composed of a series resistor and a shunt capacitor connected to ground (Figures 6A and S5). The circuit architecture and corresponding schematic are shown in Figures 6A-(a) and 6A-(b). In this configuration, low-frequency input signals pass through the resistor with minimal attenuation, while high-frequency components are progressively shunted to ground through the capacitor, resulting in frequency-dependent signal attenuation at the output. The fabrication workflow, summarized in Figure S5a, consists of five straightforward steps: (i) laser-induced hydrophilic patterning on parchment paper, (ii) screen printing of the resistive element, (iii) screen printing of interdigitated capacitor electrodes, (iv) screen printing of conductive interconnects, and (v) screen printing of the PVA-based gel electrolyte to complete the capacitor.

**Figure 6:**
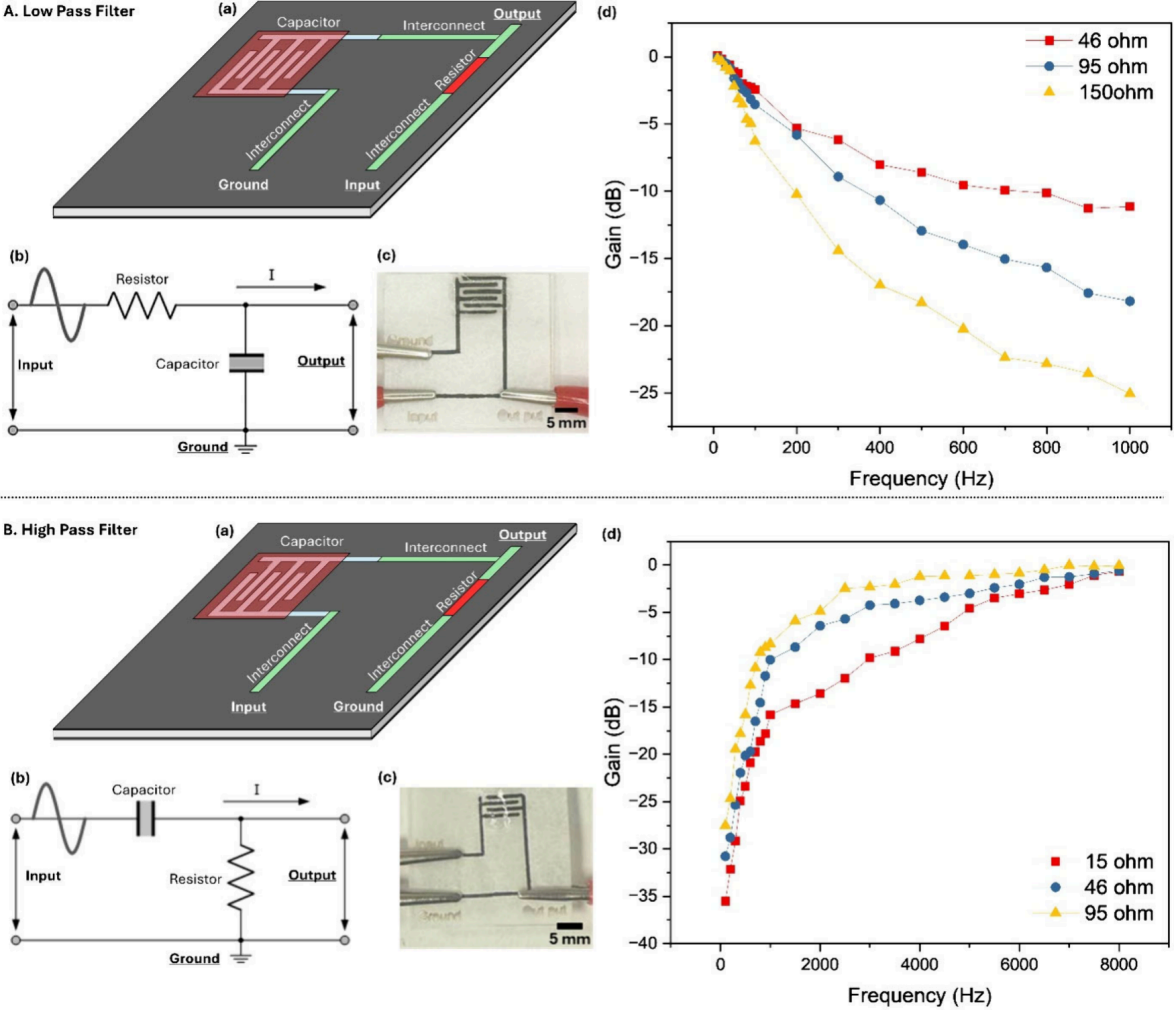
Paper-based RC filters fabricated on parchment paper. A. Low-pass RC filter: (a) schematic illustration, (b) circuit diagram, and (c) photograph of the low-pass filter fabricated on parchment paper. (d) Frequency-dependent gain plots for low-pass filters with different resistance values (46 Ω, 95 Ω, and 150 Ω), demonstrating low-pass cutoff behavior. B. High-pass RC filter: (a) schematic illustration, (b) circuit diagram, and (c) photograph of the high-pass filter fabricated on parchment paper. (d) Frequency-dependent gain plots for high-pass filters with different resistance values (15 Ω, 46 Ω, and 95 Ω), demonstrating high-pass cutoff behavior.

Because the resistor, capacitor electrodes, and interconnects require different ink formulations and additives, each printing step is performed using a dedicated paper stencil. Despite this, the overall process remains low-temperature and highly reproducible. A representative low-pass filter device fabricated on parchment paper is shown in Figure 6A-(c). Electrical characterization was first carried out on a device incorporating a 46 Ω resistor and a 28 μF capacitor. The experimentally measured frequency-dependent gain and phase responses are presented in Figure S5b and S5c, respectively. The voltage gain was calculated as Gain (dB) = 20 log_10_(V_out_/V_in_), where V_in_ is the peak-to-peak amplitude of the sinusoidal input signal (100 mV) and V_out_ is the output amplitude through a high-impedance probe (10 MΩ). The frequency was swept logarithmically, and the −3 dB cutoff frequency was defined as the frequency at which the gain decreased by 3 dB from the passband value. As expected for a first-order low-pass filter, the gain remains near 0 dB at low frequencies and decreases monotonically with increasing frequency (Figure S5b). The output signal drops below −10 dB at frequencies above approximately 600 Hz, with a measured −3 dB cutoff frequency of 112 Hz. The corresponding phase response exhibits a smooth transition from approximately 0° at low frequencies to nearly −90° at high frequencies (Figure S5c), confirming the expected phase lag behavior of a passive RC low-pass filter and validating the analog signal-processing capability of the paper-based circuit. To demonstrate tunability without altering circuit geometry or capacitor design, the resistance was systematically adjusted by varying the DMSO concentration in the PEDOT:PSS resistor ink while maintaining identical printed dimensions (5 mm length). DMSO concentrations of 10%, 8%, and 6% yielded resistances of 46 Ω, 95 Ω, and 150 Ω, respectively (Figure S5d). The capacitor value was fixed at approximately 28 μF by filling a six-finger interdigitated electrode structure with 5 μL of H_3_PO_4_-doped PVA gel electrolyte. As shown in Figure 6A­(d), increasing the resistance leads to progressively lower cutoff frequencies and stronger attenuation, in excellent agreement with theoretical predictions. The measured cutoff frequencies shift from 112 Hz (46 Ω) to 56 Hz (95 Ω) and 38 Hz (150 Ω). These values closely match the calculated cutoff frequencies of 123, 60, and 37 Hz, respectively (Figure S5e), obtained using the standard first-order relation:

3 $$f_{c}=1/2\pi RC$$

where *f*_*c*_ is the cutoff frequency, and *R* and *C* denote the resistance and capacitance values. The strong agreement between calculated and measured cutoff frequencies highlights the predictability and robustness of the laser-patterned papertronic platform. Importantly, circuit tuning is achieved exclusively through ink formulation—without modifying device layout, substrate treatment, or fabrication flow. This capability enables scalable, programmable analog circuit design directly on paper.

To extend the demonstrated circuit functionality beyond low-frequency attenuation, fully integrated high-pass RC filters were fabricated on hydrophobic parchment paper using the same laser-defined patterning and sequential ink deposition strategy employed for the low-pass circuits. This confirms that the proposed papertronic platform supports bidirectional frequency selectivity and programmable analog signal conditioning. The high-pass filters adopt a canonical first-order RC configuration in which the capacitor is placed in series with the input, while the resistor connects the output node to ground (Figures 6B-(a) and 6B-(b)). In this topology, low-frequency components are effectively blocked by the capacitive impedance, whereas higher-frequency signals are transmitted to the output. A representative fabricated high-pass filter device is shown in Figure 6B-(c), illustrating seamless integration of the capacitor, resistor, and interconnects within a compact paper-based footprint. Fabrication followed the same five-step process as the low-pass filters (Figure S6a). Separate paper stencils were used for each printing step to accommodate different ink formulations and additive concentrations while maintaining electrical isolation and component fidelity. Electrical characterization was first performed on a high-pass filter incorporating a 15 Ω resistor and a 2 μF capacitor. The frequency-dependent gain response (Figure S6b) exhibits the expected high-pass behavior, with strong attenuation at low frequencies and gradual recovery toward unity gain at higher frequencies. Specifically, the gain increases from approximately – 35 dB at 10 Hz to nearly 0 dB at ∼8 kHz, indicating effective suppression of low-frequency signals and minimal attenuation in the passband. Phase response measurements (Figure S6c) further confirm proper high-pass operation, showing a smooth transition from large phase lead at low frequencies toward near-zero phase shift at higher frequencies. This behavior is characteristic of a well-behaved first-order high-pass filter and indicates stable circuit operation without parasitic effects. To demonstrate tunability, additional filters were fabricated using 46 Ω and 95 Ω resistors while keeping the capacitance fixed at 2 μF (Figure S6d). Increasing resistance systematically shifts the cutoff frequency toward lower values. The filters with 46 Ω and 95 Ω resistors exhibited measured cutoff frequencies of 1610 and 770 Hz, respectively (Figure 6B­(d)). All measured cutoff frequencies for the different resistor values are in close agreement with the theoretical expectations summarized in Figure S6e. The frequency response trends clearly show that increasing resistance enhances low-frequency attenuation and shifts the transition region downward, confirming that the RC time constant can be precisely tuned through ink formulation alone, without modifying circuit geometry or substrate treatment. Together with the low-pass filters, these results establish laser-induced papertronics as a robust platform for integrated analog circuits on paper, in which frequency-selective behavior is programmed through ink formulation alone.

### 2.5 Degradability, Disposability, and Packaging of Papertronics

Having previously demonstrated biodegradation in cellulose-based papertronics,^40^ we extend this evaluation to silicone-coated parchment paper. Although parchment paper incorporates a thin silicone coating to impart hydrophobicity, the substrate remains predominantly cellulose-based, with the silicone layer accounting for only a minor fraction of the total material volume (typically <2 μm in thickness). As such, degradation is governed primarily by the underlying cellulose fiber network. The silicone coating, while more persistent in natural environments, is widely recognized as biocompatible and chemically inert, and its limited mass minimizes its environmental burden.^41,60^ To evaluate degradation behavior, individual paper-based electronic components (resistors and capacitors) as well as integrated RC filter circuits were subjected to degradation studies under three conditions: (i) natural soil in a controlled laboratory environment (Figure 7a), (ii) natural soil under outdoor environmental exposure (Figure 7b), and (iii) a bacterial culture containing *Shewanella oneidensis* MR-1 (Figure 7c). Visual inspection reveals progressive fragmentation, fiber delamination, and loss of structural integrity over time in all cases. After 4 weeks of soil burial under either controlled laboratory conditions or outdoor environmental exposure, the majority of the cellulose-based regions had disintegrated and assimilated into the surrounding soil, leaving only small residual fragments (Figure 7a and 7b). Even in bacterial culture (Figure 7c), biodegradation of the devices was clearly observed, with progressive structural disintegration over time. Quantitative weight-loss measurements further support these observations (Figure 7d). After 4 weeks, the resistor, capacitor, and RC filter circuit exhibited mass losses exceeding 25%, ∼22%, and ∼27%, respectively. Notably, degradation of the capacitor proceeded slightly more slowly than that of the resistor, which can be attributed to the presence of the PVA-based gel electrolyte that degrades more slowly than the predominantly PEDOT:PSS-based resistive layer. In contrast, the RC filter circuit showed the highest overall mass loss due to its larger exposed paper area, which accelerates microbial access and enzymatic degradation. It should be noted that the degradation data presented here demonstrates physical disintegration and mass loss under environmentally relevant conditions, which are necessary but not sufficient indicators of complete biodegradation. A comprehensive assessment of degradation byproducts and their ecotoxicological profiles was not performed in this study and remains an important direction for future work. However, all functional materials used in these devices—including PEDOT:PSS, PVA, H_3_PO_4_, and trace quantities of graphene and MXene—are drawn from material classes with well-established biocompatibility and low environmental toxicity in the published literature.^40,61^ The cellulose substrate, which constitutes the vast majority of the device mass, undergoes well-characterized enzymatic biodegradation to nontoxic products (glucose, CO_2_, H_2_O). The silicone coating, while more environmentally persistent, is present at minimal mass (<2 μm layer) and is widely recognized as chemically inert and biocompatible.^60^ These considerations provide supporting evidence that the degradation products are unlikely to pose significant environmental risk at the quantities relevant to disposable paper-based electronics, though dedicated ecotoxicological studies are warranted for regulatory-level assessment. Compared with wax-patterned devices,^40^ parchment-based papertronics showed comparable or slightly improved biodegradation, as the ultrathin silicone coating (<2 μm) impedes microbial access far less than bulk paraffin wax barriers.

**Figure 7:**
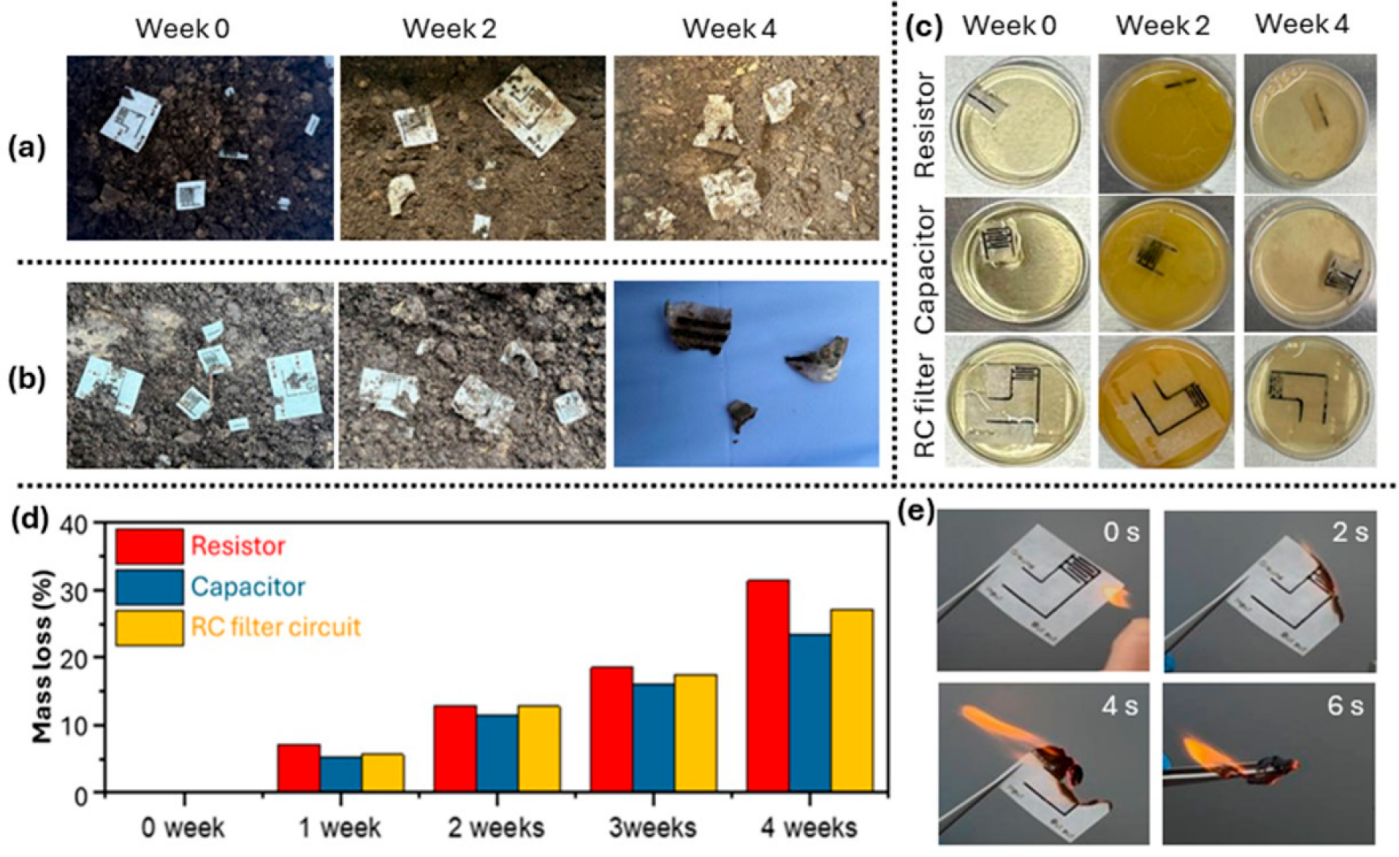
Biodegradability and disposability assessment of paper-based electronic components and integrated circuits. Biodegradation dynamics: Sequential photographs showing the degradation of individual paper-based electronic components (resistor and capacitor) as well as an integrated RC filter circuit in (a) natural soil under laboratory conditions, (b) natural soil under outdoor environmental exposure, and (c) a bacterial culture medium. (d) Quantitative weight-loss analysis of the devices as a function of degradation time in the bacterial culture medium. (e) Burnability test: Time-lapse images of the combustion of the RC filter circuit, captured at 2 s intervals, documenting complete combustion within 6 s.

Beyond biological and environmental degradation, incineration represents a rapid and practical disposal pathway for transient electronics. Figure 7e shows a time-lapse sequence of an RC filter circuit subjected to open-flame ignition. The device undergoes complete combustion within 6 s, leaving minimal residual ash. The primary combustion products of the predominantly cellulose-based device are CO_2_ and H_2_O, with trace inorganic residues (SiO_2_ from the silicone coating, TiO_2_ from MXene) that are chemically inert and nontoxic. This qualitative demonstration highlights a practical advantage of paper-based electronics: even when coated with silicone and functional inks, the overall material system remains readily combustible and amenable to rapid volume reduction, though quantitative emissions characterization under controlled conditions remains an important direction for formal waste-management assessment. Such incineration-based disposal is particularly relevant for short-lifetime electronics, environmental sensors, and disposable diagnostic platforms, where rapid and controlled waste elimination is desirable. Importantly, the thin silicone coating does not prevent combustion, underscoring that parchment-based papertronics remain compatible with incineration as an end-of-life strategy.

While degradability and disposability are central to sustainable papertronics, packaging remains a critical and largely underexplored challenge. Paper substrates are inherently vulnerable to mechanical damage, humidity-induced swelling, and premature degradation, all of which can degrade electrical performance and shorten operational lifetime.^34,40^ Effective encapsulation strategies are therefore essential for extending device usability without undermining sustainability. In this work, PDMS was selected as an encapsulation material due to its widespread use in flexible and organic electronics, stemming from its flexibility, hydrophobicity, optical transparency, biocompatibility, and ease of processing.^60,62^ PDMS is particularly effective at suppressing moisture ingress and mitigating mechanical stresses, making it well suited for protecting paper-based devices during handling and operation.^63^ Encapsulation was achieved by gently dispensing PDMS onto the device surface and spreading it uniformly using a blade to form a thin conformal layer (∼300 μm). Electrical contact pads were masked prior to coating to preserve electrical access. An identical PDMS layer was subsequently applied to the backside of the paper substrate, creating a sandwich-type encapsulation that maintains overall flexibility while providing environmental isolation. The impact of encapsulation on circuit performance was evaluated by comparing low-pass and high-pass RC filters before and after PDMS coating under identical measurement conditions (Figure S7). The frequency response curves exhibit nearly identical gain profiles and cutoff frequencies, indicating that PDMS encapsulation does not interfere with analog circuit operation. For example, the low-pass filter (46 Ω, 28 μF) retained a cutoff frequency of ∼112 Hz before encapsulation and ∼109 Hz after encapsulation, while the high-pass filter (15 Ω, 2 μF) showed only a minor shift from 5230 to 5190 Hz. These differences fall within experimental variation and confirm electrical stability. While these results demonstrate effective short-term environmental protection, long-term accelerated aging studies under controlled humidity and temperature cycling were not performed in this work, as the devices are primarily intended for short-lifetime, disposable applications. Systematic evaluation of shelf life and operational longevity under standardized environmental conditions (e.g., 85 °C/85% RH) represents an important future milestone for applications requiring extended operational lifetimes.

## 3 Future Direction

Building on these results, several directions can further expand laser-enabled papertronics. Beyond demonstrating improved patterning fidelity, this study validates the platform through fully functional passive components and integrated analog circuits, confirming its immediate relevance for practical paper-based electronics. Building on this foundation, several strategic directions emerge to further expand the scope and impact of laser-enabled papertronics. First, the extension of this patterning approach to a broader library of electronic components—including inductors, diodes, and active devices such as transistors—will be critical for enabling more sophisticated circuit architectures. Many of these components are inherently compatible with solution-processable materials and could be integrated using the same laser-defined confinement strategy demonstrated here. Second, multilayer integration represents a key opportunity for advancing circuit complexity and functional density. By combining laser-patterned layers with vertical interconnects and insulating interlayers, paper-based electronics could transition from planar demonstrations to PCB-like stacked architectures, significantly enhancing performance while maintaining mechanical flexibility and sustainability. The hydrophobic parchment substrate naturally serves as an interlayer insulator in stacked configurations, and its foldability—previously exploited in our multilayer wax-based PCBs^40^—is fully preserved after laser treatment, providing a direct pathway to folded or stacked circuit architectures. Such multilayer configurations would enable compact signal routing, reduced footprint, and improved circuit robustness. Third, coupling laser-induced patterning with hybrid material systems, including semiconducting polymers, bioderived conductors, and emerging two-dimensional materials, could unlock new device functionalities while preserving the environmental advantages of paper substrates. Integration with sensing, energy harvesting, and transient electronics platforms offers additional pathways toward deployable systems for environmental monitoring, disposable diagnostics, and low-cost IoT nodes. Additionally, comprehensive ecotoxicological characterization of degradation byproducts—including respirometric analysis, dissolved organic carbon tracking, and standardized aquatic toxicity assays—will be essential for establishing the environmental safety credentials required for regulatory acceptance of disposable papertronics. Finally, additional gains in resolution and circuit density are expected through optical optimization of the laser system. While the present work employed a standard 2-in. focusing lens, the use of high-power-density focusing optics (HPDFO)—capable of reducing the laser spot size to approximately one-quarter to one-fifth of that achieved with a 2-in. lens—offers a clear pathway toward further miniaturization. Such optics could realistically enable hydrophilic features on the order of ∼60 μm (theoretical) and ∼100 μm (practical), corresponding to a two- to 5-fold improvement in lateral resolution. This advancement would directly translate to higher circuit density, finer interconnect pitch, and expanded compatibility with more complex electronic designs. Together, these pathways can position laser-patterned paper as a competitive platform for targeted applications in sustainable electronics.

## 4 Conclusion

This work introduces a laser-induced hydrophilic patterning strategy on hydrophobic parchment paper as a scalable route to high-density papertronics. By eliminating wax-based barriers, the approach enables sharply defined hydrophilic features, improved lateral resolution, and deterministic ink confinement. Fully printed resistors, interconnects, capacitors, and integrated low- and high-pass RC filters were demonstrated using solution-processable functional inks. The improved patterning resolution enables a materials-programmable design approach in which resistance, capacitance, and filter cutoff frequency are tuned over wide ranges through ink formulation alone—without modifying device geometry or substrate treatment—yielding predictable electrical behavior consistent with circuit theory. The devices maintain biodegradability and disposability while benefiting from optional PDMS encapsulation for enhanced robustness. Taken together, these results establish laser-patterned parchment paper as a versatile, sustainable platform for integrated paper-based electronic systems.

### Materials and Methods

#### Laser Patterning

All laser-patterned papertronic components, including passive elements and integrated RC filter circuits, were fabricated on commercial silicone-coated parchment paper (10 × 10 in, 35#, Silicone Treated Heavy Duty Parchment Paper Squares, Amazon). This substrate was selected for its smooth surface, dimensional stability, appropriate thickness (∼75 μm), and low intrinsic porosity, which together enable high-resolution patterning and reliable ink confinement. This commercial substrate is manufactured under industrial quality-control standards for the food-packaging industry, providing consistent silicone coating thickness, paper weight, and surface smoothness across production batches. Multiple sheets from different packages were used throughout this study, and no systematic differences in patterning quality or electrical performance were observed. A systematic evaluation of patterning sensitivity to different commercial parchment products with varying silicone chemistries and coating thicknesses represents an interesting direction for future optimization. A 50 W CO_2_ laser cutter (VLS 3.50 laser system) was used to define hydrophilic microchannels by locally modifying the surface energy of the hydrophobic parchment paper. Device layouts were designed using AutoCAD and exported via CorelDRAW to ensure compatibility with the laser system interface. To achieve high patterning resolution while avoiding thermal damage to the underlying cellulose matrix, the laser power and scan speed were systematically optimized. The optimized laser parameters were identified to be approximately 10–12% power at 100% scan speed, which consistently produced well-defined, continuous hydrophilic features suitable for subsequent ink-guided material deposition. Importantly, no additional chemical treatments or postprocessing steps were required following laser exposure, thereby preserving the mechanical integrity, flexibility, and surface uniformity of the parchment substrate.

#### Fabrication of Resistors

Resistors were designed with a simple rectangular geometry and systematically varied lengths to enable predictable resistance tuning and straightforward electrical interfacing with external circuits. This geometry also facilitates quantitative comparison of resistive behavior across devices. The conductive ink used for resistor fabrication consisted of PEDOT:PSS blended with DMSO. PEDOT:PSS was diluted in deionized water to concentrations ranging from 50 to 100 wt %, while the DMSO content was varied from 2 to 20 wt %. This formulation allowed exploration of a wide range of resistive properties through controlled modification of both polymer concentration and secondary doping level. Prior to ink deposition, parchment paper substrates were laser-patterned to define hydrophilic rectangular channels surrounded by hydrophobic regions, which served as effective barriers to confine the conductive ink and ensure consistent resistor geometry. Following laser patterning, the PEDOT:PSS/DMSO ink was initially dispensed using a micropipette into channels with lengths of 5, 10, 15, and 20 mm, while maintaining a constant channel width (800 μm) to standardize resistance calculations and enable direct comparison across devices. To further improve film uniformity and thickness control, the deposited ink was subsequently screen-printed using a blade, which evenly redistributed the ink within the laser-defined region. Owing to the smooth surface of the parchment paper, this blade-assisted screen-printing step also removed excess ink residues and produced clean, well-defined resistor patterns with uniform coverage. The patterned resistors were then allowed to dry overnight at ambient conditions without thermal treatment, preserving the mechanical integrity and dimensional stability of the paper substrate.

#### Fabrication of Interconnects

Interconnects were fabricated using a process analogous to that employed for resistors, but with design and material modifications aimed at achieving low resistance over extended lengths, as required for reliable signal and power delivery in integrated circuits. Unlike resistors—where resistance tunability is desirable—interconnects were engineered to minimize electrical resistance while maintaining mechanical flexibility and environmental stability. Laser-defined hydrophilic channels were first patterned on hydrophobic parchment paper to confine conductive inks with high spatial precision. A constant channel width of 800 μm was used to ensure a sufficiently large conductive cross-section, while the interconnect length was systematically extended up to several centimeters, representing an extreme case for paper-based wiring. This design allowed assessment of conductivity preservation over long distances without sacrificing pattern fidelity. To further enhance electrical conductivity and improve mechanical and thermal robustness, two composite conductive ink formulations were developed. The first consisted of PEDOT:PSS blended with 5 wt % graphene and 20 wt % DMSO, while the second replaced DMSO with 20 wt % Ti_3_C_2_T_*x*_ MXene. Graphene was incorporated to increase charge transport pathways through its high aspect ratio and excellent electrical conductivity, while also reinforcing the polymer matrix. DMSO was used as a secondary dopant in the first formulation to enhance PEDOT chain ordering and reduce the insulating PSS fraction, whereas MXene was employed in the second formulation to introduce highly conductive two-dimensional flakes capable of improving electrical continuity and mechanical durability without the need for solvent-based secondary doping. Both ink formulations were prepared by probe sonication for 2 h, ensuring homogeneous dispersion of graphene and MXene nanosheets within the PEDOT:PSS matrix and preventing aggregation that could compromise printability or electrical uniformity. Following laser-induced hydrophilic patterning, the inks were first introduced into the channels via micropipette dispensing to ensure complete filling. This was followed by blade-assisted screen printing. After deposition, the interconnects were allowed to dry under ambient conditions.

#### Fabrication of Capacitors

Paper-based capacitors were fabricated using an in-plane interdigitated electrode architecture, which enables efficient charge storage while minimizing device footprint and allowing precise geometric control over capacitance. This planar configuration is particularly well suited for paper substrates, as it avoids the need for through-thickness electrolyte penetration and complex multilayer alignment that often limit reproducibility in vertical paper capacitors. Each capacitor consisted of multiple interdigitated electrode “fingers” patterned on hydrophobic parchment paper with laser-defined hydrophilic regions. Devices incorporating 4, 6, 8, and 10 electrode fingers were designed to systematically investigate the effect of electrode density and effective interfacial area on capacitance (Figure S4). The laser-induced hydrophilic patterning ensured sharp electrode boundaries and prevented lateral spreading of conductive ink, enabling reproducible feature definition even at high finger densities. The interdigitated electrodes were formed using a conductive composite ink composed of PEDOT:PSS blended with 20 wt % DMSO and 5 wt % graphene. Electrode patterns were defined by precision micropipette deposition within laser-activated hydrophilic regions, followed by blade-assisted spreading to ensure uniform coverage across the electrode fingers. The electrode geometry was kept constant across all devices, with a finger length of approximately 1 mm, finger width of ∼800 μm, and interfinger spacing of ∼ 800 μm, allowing capacitance variations to be attributed primarily to electrode number and electrolyte composition. The printed electrodes were dried under ambient conditions at room temperature to avoid thermal deformation or shrinkage of the parchment substrate. To form the dielectric and ionic conduction medium, an aqueous PVA-based gel electrolyte was deposited directly onto the interdigitated electrode array. The PVA gel was prepared by dissolving 1 g of PVA in deionized water under continuous stirring at 90 °C for 1 h, producing a homogeneous viscous solution. After cooling to room temperature, phosphoric acid (H_3_PO_4_) was added in controlled volumes to tune the ionic conductivity and dielectric properties of the electrolyte. The concentration of H_3_PO_4_ directly modulates ion mobility and electric double-layer formation at the electrode–electrolyte interface, thereby providing a powerful handle for capacitance tuning. The resulting PVA/H_3_PO_4_ gel electrolyte was selectively deposited onto the electrode region using a laser-patterned paper stencil, ensuring uniform coverage of the interdigitated fingers while preventing electrolyte overflow into surrounding regions. Following deposition, the devices were allowed to rest and dry overnight under ambient conditions, yielding mechanically stable capacitors with well-adhered gel electrolytes and preserved electrode geometry. The capacitive performance of each device was systematically characterized to correlate electrode geometry (finger number) and electrolyte composition (H_3_PO_4_ concentration) with the resulting capacitance values. This approach enabled reliable and reproducible tuning of capacitance across the microfarad-to-millifarad range while maintaining a simple, low-temperature, and wax-free fabrication workflow.

#### Fabrication of RC Filters

Fully printed first-order RC low-pass and high-pass filters were fabricated directly on silicone-coated parchment paper by combining laser-induced hydrophilic patterning with sequential introduction of component-specific functional inks. Each circuit integrated a resistor, interconnects, and an interdigitated capacitor within a single paper layer, forming compact analog signal-processing units without the need for multilayer stacking or external assembly. Both low-pass and high-pass filters were realized using the same fundamental fabrication workflow, differing only in circuit topology and ink placement. In each case, the laser was first used to define hydrophilic microchannels that served as deterministic templates for ink confinement. This single-step, mask-free laser modification enabled precise definition of resistive elements, capacitor electrodes, and interconnect pathways, ensuring reproducible device geometry and preventing lateral ink spreading—a critical limitation in conventional wax-patterned paper circuits. The complete fabrication of each RC filter required only five sequential steps, as detailed in the main manuscript:(i) laser-induced hydrophilic patterning of the circuit layout on parchment paper; (ii) screen-printing of conductive ink to form the resistor; (iii) screen-printing of conductive ink to define the interdigitated capacitor electrodes; (iv) screen-printing of conductive ink for interconnect formation; and (v) deposition of the PVA-based gel electrolyte to complete the capacitor (Figure S5a and S6a). Because resistors, capacitor electrodes, and interconnects require different ink formulations and electrical properties, separate paper stencils were used for each printing step to maintain material selectivity and device integrity.

Following circuit fabrication, the paper-based RC filters were encapsulated in PDMS to enhance mechanical robustness and environmental stability, particularly against humidity, handling damage, and repeated bending. PDMS (Sylgard 184) was prepared by mixing the base and curing agent at a 10:1 weight ratio, followed by thorough stirring to ensure homogeneous mixing. The mixture was then degassed under vacuum for approximately 10–20 min to remove trapped air bubbles. Encapsulation was performed by drop casting followed by blade coating, forming a thin and conformal PDMS layer with a thickness of approximately 300 μm over the circuit surface. To further enhance environmental protection, a second PDMS layer was applied to the backside of the paper using the same procedure, resulting in a sandwiched encapsulation structure while maintaining overall flexibility. To preserve the structural integrity of the paper substrate and prevent ink delamination or electrolyte dehydration, PDMS curing was carried out at room temperature for 24 h, avoiding elevated-temperature processing. Electrical contact pads were protected during coating using removable masking materials, which were removed after curing to allow electrical access. After encapsulation, the RC filters were electrically characterized to assess frequency response, cutoff behavior, and phase characteristics.

#### Electrical Characterization

The electrical performance of individual components and integrated circuits was systematically characterized using standard benchtop instrumentation. The conductance and resistance of resistors and interconnects were measured using a Lomvum T28B digital multimeter, complemented by four-point probe measurements to minimize contact resistance effects and ensure accurate determination of intrinsic material properties. Capacitors were initially evaluated using the digital multimeter to obtain static capacitance values, followed by detailed electrochemical characterization using a potentiostat/galvanostat (Admiral Squidstat Plus). GCD measurements were performed at controlled current levels to extract capacitance, internal resistance, and cycling stability. Capacitance values were calculated from the discharge profiles, accounting for voltage drop associated with internal resistance. For integrated RC filter circuits, sinusoidal input signals were generated using a function generator (Tektronix AFG3022B), and output signals were recorded with a digital oscilloscope (Keysight MSO-X 3054A) equipped with a high-impedance probe (10 MΩ) to avoid circuit loading effects. Frequency-dependent gain and phase responses were extracted across a broad frequency range to evaluate filter behavior, cutoff frequencies, and circuit stability before and after encapsulation.

#### Optical and Morphological Analysis

Optical and morphological characterization of the paper-based electronic components was conducted to examine ink confinement, cross-sectional structure, and material interfaces. For cross-sectional analysis, samples were first cryogenically frozen in liquid nitrogen and then fractured at their midpoint to obtain clean and representative cross sections without smearing or fiber deformation. The fractured samples were examined using a V12 stereo optical microscope for low-magnification inspection and a Hitachi SU5000 field-emission scanning electron microscope (FE-SEM) for high-resolution imaging. SEM analysis enabled visualization of conductive ink penetration into the cellulose fiber network, verification of sharp hydrophilic–hydrophobic boundaries, and confirmation of layer integrity for resistors, interconnects, and capacitors.

#### Degradation Test

The degradability of paper-based electronic components and integrated RC filter circuits was evaluated under three complementary conditions to assess environmental relevance and microbial effects: 1. Natural soil under controlled laboratory conditions, maintained at 25 °C and 60% relative humidity; 2. Natural soil under outdoor environmental exposure, enabling assessment under fluctuating temperature and humidity conditions (Figure 7b); 3. Bacterial degradation using *Shewanella oneidensis* MR-1, a model electroactive microorganism known for its extracellular electron transfer capabilities (Figure 7c). Soil samples were collected on the campus of the State University of New York at Binghamton in July 2025. The outdoor soil burial tests provided field-level validation of biodegradation behavior, complementing the controlled laboratory experiments. For bacterial degradation studies, devices were immersed in 10 mL of Luria–Bertani (LB) medium inoculated with *S. oneidensis* at an optical density (OD_600_) of 2.0. To sustain microbial activity and consistent degradation conditions, the bacterial culture was refreshed daily with fresh LB medium. Biodegradation progress was quantitatively monitored by weekly mass-loss measurements. Prior to weighing, samples were rinsed thoroughly with deionized water to remove residual medium and biomass, then dried to constant weight to ensure accurate mass determination.

#### Statistical Analysis

All experiments were conducted using a minimum of three independent replicates. Data are presented as mean ± standard error (SE) unless otherwise stated. Statistical analysis and graphical visualization were performed using Origin software (OriginLab, USA).

## Supplementary Material



## Notes

S1: Optimization of laser treatment on parchment paper. Laser patterning performed at a constant scan speed of 100% with varying laser power for (i–iv) straight-line patterns and (v) circular patterns. Laser patterning with different line widths and line spacings to evaluate achievable resolution (unit: mm). Laser-treated parchment paper designed to produce 200 μm line width and 200 μm spacing features. S2: Cross-conductivity measurements. Conductivity tests performed between adjacent straight conductive lines and along a single conductive line. The individual line width and line spacing are 250 and 300 μm, respectively. Conductive lines were fabricated using PEDOT:PSS as the sole conductive ink, without additional fillers or dopants. S3: FTIR analysis of parchment paper before and after laser treatment and PEDOT:PSS introduction. FTIR spectra of untreated parchment paper, laser-treated parchment paper, and laser-treated parchment paper with patterned PEDOT:PSS. S4: Paper-based Capacitors. Schematic illustration of a capacitor array fabricated on paper. Photographs of fabricated capacitors with different numbers of interdigitated electrode fingers, including device dimensions. S5: Low-pass RC filter fabricated on parchment paper. Fabrication process of the low-pass filter on parchment paper. Frequency-dependent gain plot of the low-pass filter with a 46 Ω resistor, demonstrating low-pass cutoff behavior. Corresponding phase shift as a function of frequency, indicating the characteristic phase lag introduced by the RC circuit. Component details, including capacitance and resistance values, device dimensions, and ink compositions. Comparison of calculated and experimentally measured cutoff frequencies for varying resistor values with a fixed 28 μF capacitor. S6: High-pass RC filter fabricated on parchment paper. Fabrication process of the high-pass filter on parchment paper. Frequency-dependent gain plot of the high-pass filter with a 15 Ω resistor, demonstrating high-pass cutoff behavior. Corresponding phase shift as a function of frequency, indicating the characteristic phase response introduced by the RC circuit. Component details, including capacitance and resistance values, device dimensions, and ink compositions. Comparison of calculated and experimentally measured cutoff frequencies for varying resistor values using a fixed 2 μF capacitor. S7: PDMS encapsulation of papertronic RC filters. A. Low-pass RC filter: Photographs of low-pass filter papertronic devices without and with PDMS encapsulation. Frequency-dependent gain plots of the low-pass filters before and after PDMS encapsulation using a 46 Ω resistor, demonstrating preserved low-pass cutoff behavior with comparable performance. B. High-pass RC filter: Photographs of high-pass filter papertronic devices without and with PDMS encapsulation. Frequency-dependent gain plots of the high-pass filters before and after PDMS encapsulation using a 15 Ω resistor, demonstrating preserved high-pass cutoff behavior with comparable performance.(PDF)
