# Supplementary material for: High-Density Papertronics via Laser-Written Hydrophilicity on Hydrophobic Parchment Paper
Source: ACS Appl Mater Interfaces. 2026 Apr 16;18(16):23472–90. doi: 10.1021/acsami.6c03065 (PMC13133782; doi:10.1021/acsami.6c03065)
Supplement: Supplementary file 1 [file am6c03065_si_001.pdf]

Supporting Information

# High-Density Papertronics via Laser-Written Hydrophilicity on Hydrophobic Parchment Paper

*Zahra Rafiee[1], Ruohan Zhang[1], and Seokheun Choi[1,2]**

[1]Bioelectronics & Microsystems Laboratory, Department of Electrical & Computer Engineering, State University of New York at Binghamton, Binghamton, New York, 13902, USA

[2]Center for Research in Advanced Sensing Technologies & Environmental Sustainability, State University of New York at Binghamton, Binghamton, New York, 13902, USA

*Corresponding Author. Email: sechoi@binghamton.edu

Lab website: http://ws.binghamton.edu/choi/
Center website: http://www.ws.binghamton.edu/creates/

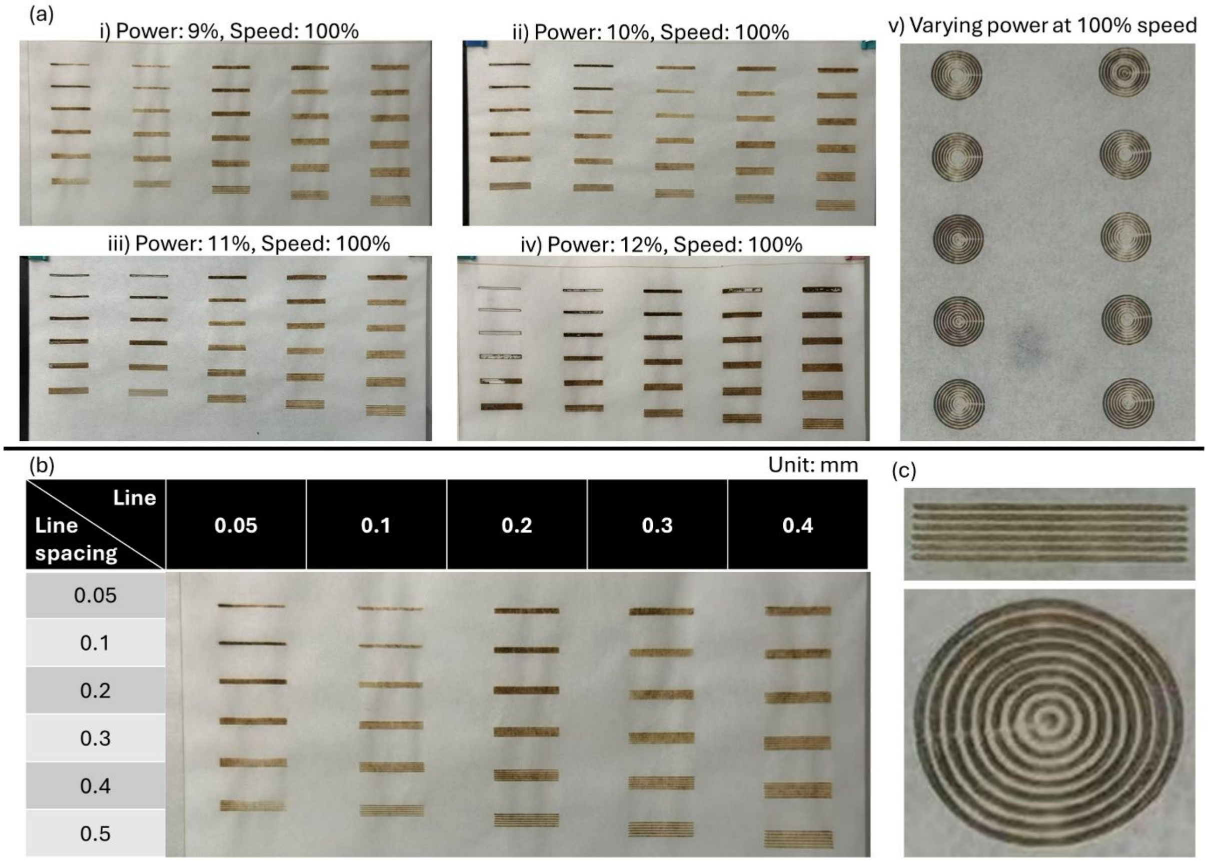


Figure S1. Optimization of laser treatment on parchment paper. (a) Laser patterning performed at a constant scan speed of 100% with varying laser power for (i–iv) straight-line patterns and (v) circular patterns. (b) Laser patterning with different line widths and line spacings to evaluate achievable resolution (unit: mm). (c) Laser-treated parchment paper designed to produce 200 μm line width and 200 μm spacing features.

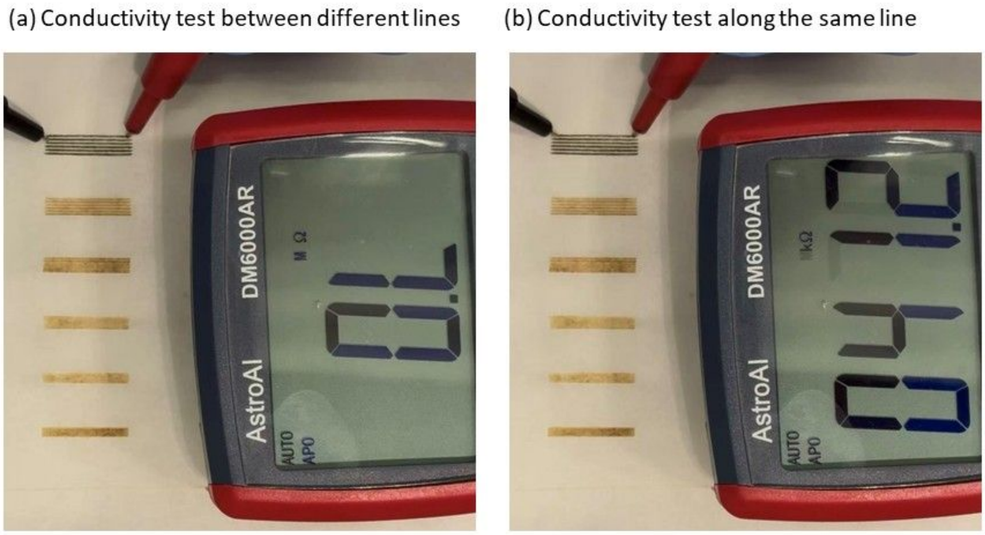


Figure S2. Cross-conductivity measurements. Conductivity tests performed (a) between adjacent straight conductive lines and (b) along a single conductive line. The individual line width and line spacing are 250 μm and 300 μm, respectively. Conductive lines were fabricated using PEDOT:PSS as the sole conductive ink, without additional fillers or dopants.

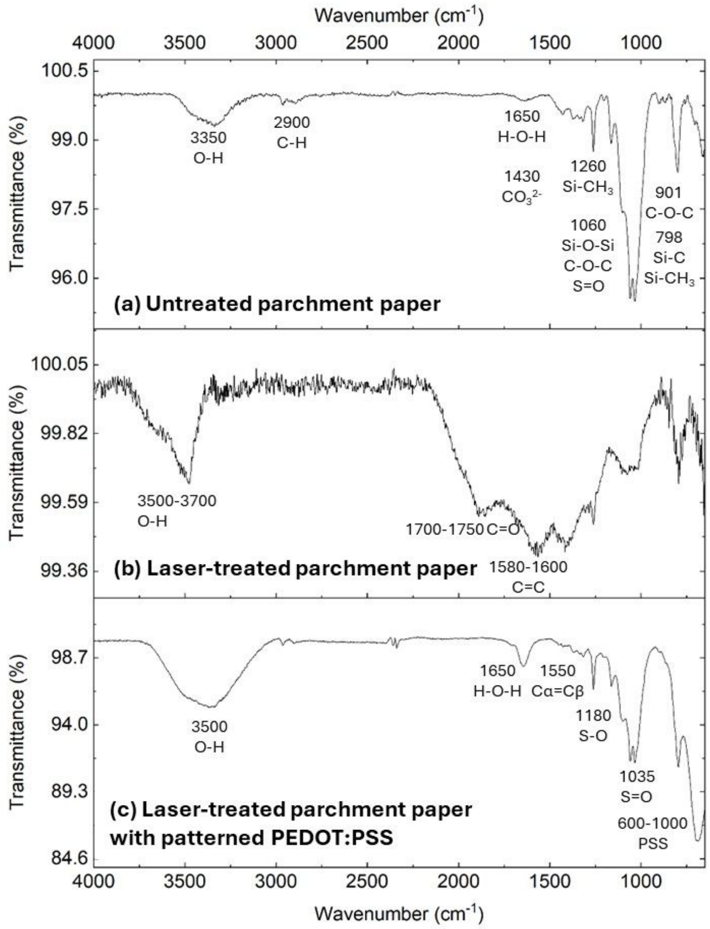


Figure S3. FTIR analysis of parchment paper before and after laser treatment and PEDOT:PSS introduction. FTIR spectra of (a) untreated parchment paper, (b) laser-treated parchment paper, and (c) laser-treated parchment paper with patterned PEDOT:PSS.

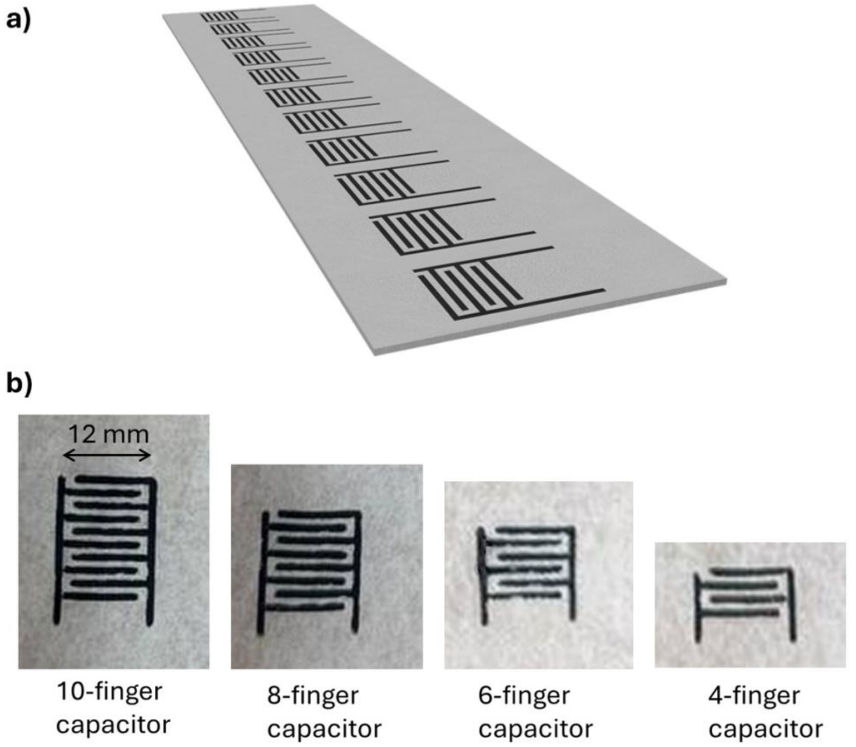


Figure S4. Paper-based Capacitors. (a) Schematic illustration of a capacitor array fabricated on paper. (b) Photographs of fabricated capacitors with different numbers of interdigitated electrode fingers, including device dimensions.

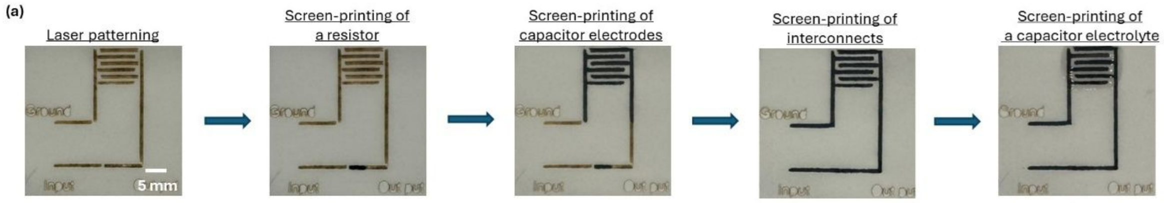


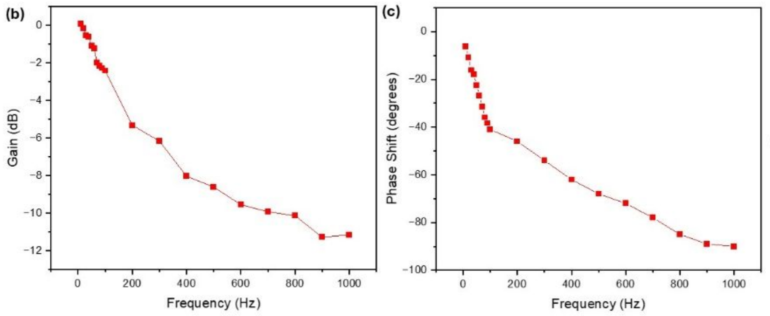


(d)

| Components | Value | Size | Material |
|---|---|---|---|
| Capacitor | 28 μF | 6 Fingers | 5ul $H_3PO_4$ |
| Resistor | 46 Ω | 5 mm | 10% DMSO |
| Resistor | 95 Ω | 5 mm | 8% DMSO |
| Resistor | 150 Ω | 5 mm | 6% DMSO |

(e)

| Capacitor (μF) | Resistor (Ω) | Calculated Cutoff Frequency (Hz) | Measured Cutoff Frequency (Hz) |
|---|---|---|---|
| 28 | 46 | 123 | 112 |
| 28 | 95 | 60 | 56 |
| 28 | 150 | 40 | 38 |

Figure S5. Low-pass RC filter fabricated on parchment paper. (a) Fabrication process of the low-pass filter on parchment paper. (b) Frequency-dependent gain plot of the low-pass filter with a 46 Ω resistor, demonstrating low-pass cutoff behavior. (c) Corresponding phase shift as a function of frequency, indicating the characteristic phase lag introduced by the RC circuit. (d) Component details, including capacitance and resistance values, device dimensions, and ink compositions. (e) Comparison of calculated and experimentally measured cutoff frequencies for varying resistor values with a fixed 28 μF capacitor.

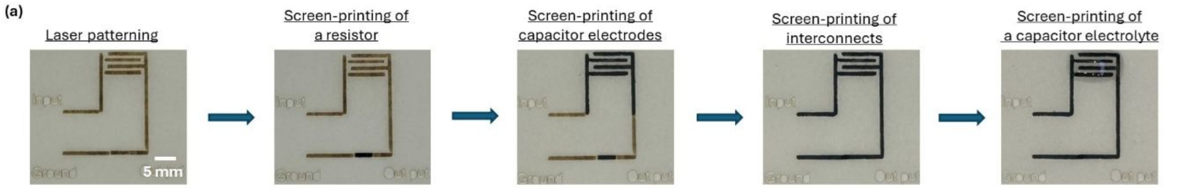


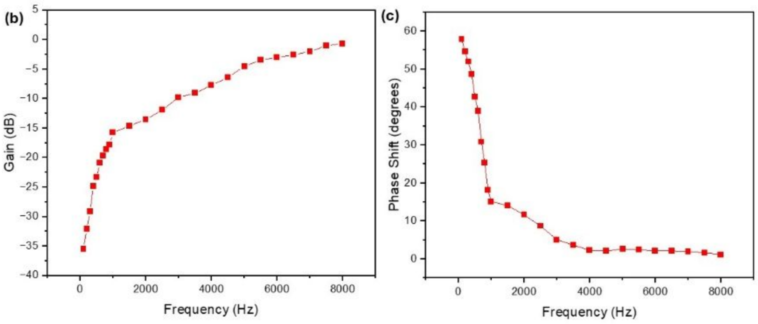


(d)

| Component | Value | Size | Material |
|---|---|---|---|
| Capacitor | 2 μF | 4 Fingers | 5ul $H_3PO_4$ |
| Resistor | 15 Ω | 5 mm | 20% DMSO |
| Resistor | 46 Ω | 5 mm | 10% DMSO |
| Resistor | 95 Ω | 5 mm | 8% DMSO |

(e)

| Capacitor (μF) | Resistor (Ω) | Calculated Cutoff Frequency (Hz) | Measured Cutoff Frequency (Hz) |
|---|---|---|---|
| 2 | 15 | 5300 | 5230 |
| 2 | 46 | 1700 | 1610 |
| 2 | 95 | 840 | 770 |

Figure S6. High-pass RC filter fabricated on parchment paper. (a) Fabrication process of the high-pass filter on parchment paper. (b) Frequency-dependent gain plot of the high-pass filter with a 15 Ω resistor, demonstrating high-pass cutoff behavior. (c) Corresponding phase shift as a function of frequency, indicating the characteristic phase response introduced by the RC circuit. (d) Component details, including capacitance and resistance values, device dimensions, and ink compositions. (e) Comparison of calculated and experimentally measured cutoff frequencies for varying resistor values using a fixed 2 μF capacitor.

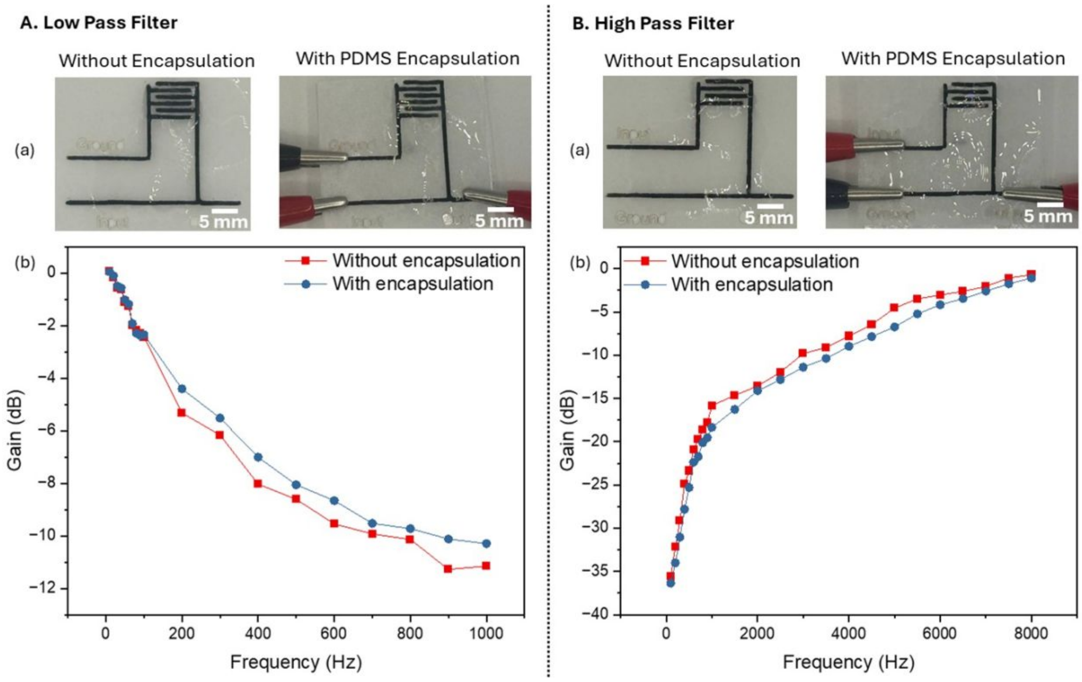


Figure S7. PDMS encapsulation of papertronic RC filters. A. Low-pass RC filter: (a) Photographs of low-pass filter papertronic devices without and with PDMS encapsulation. (b) Frequency-dependent gain plots of the low-pass filters before and after PDMS encapsulation using a 46 Ω resistor, demonstrating preserved low-pass cutoff behavior with comparable performance. B. High-pass RC filter: (a) Photographs of high-pass filter papertronic devices without and with PDMS encapsulation. (b) Frequency-dependent gain plots of the high-pass filters before and after PDMS encapsulation using a 15 Ω resistor, demonstrating preserved high-pass cutoff behavior with comparable performance.
